# Comparative analysis of genome maintenance genes in naked mole rat, mouse, and human

**DOI:** 10.1111/acel.12314

**Published:** 2015-01-28

**Authors:** Sheila L MacRae, Quanwei Zhang, Christophe Lemetre, Inge Seim, Robert B Calder, Jan Hoeijmakers, Yousin Suh, Vadim N Gladyshev, Andrei Seluanov, Vera Gorbunova, Jan Vijg, Zhengdong D Zhang

**Affiliations:** 1 Department of Genetics, Albert Einstein College of MedicineBronx, NY, 10461, USA; 2 Division of Genetics, Department of Medicine, Brigham and Women's Hospital, Harvard Medical SchoolBoston, MA, 02115, USA; 3 Department of Genetics, Erasmus University Medical CenterRotterdam, The Netherlands; 4 Department of Biology, University of RochesterRochester, NY, 14627, USA

**Keywords:** aging, gene duplication, genome maintenance, longevity, mutation rate

## Abstract

Genome maintenance (GM) is an essential defense system against aging and cancer, as both are characterized by increased genome instability. Here, we compared the copy number variation and mutation rate of 518 GM-associated genes in the naked mole rat (NMR), mouse, and human genomes. GM genes appeared to be strongly conserved, with copy number variation in only four genes. Interestingly, we found NMR to have a higher copy number of *CEBPG*, a regulator of DNA repair, and *TINF2*, a protector of telomere integrity. NMR, as well as human, was also found to have a lower rate of germline nucleotide substitution than the mouse. Together, the data suggest that the long-lived NMR, as well as human, has more robust GM than mouse and identifies new targets for the analysis of the exceptional longevity of the NMR.

## Introduction, results, discussion

DNA can be damaged by a myriad of exogenous and endogenous genotoxic agents, making GM an essential defense system. GM is complex, requiring multiple, coordinated cellular activities, including DNA repair, cellular senescence, and apoptosis. These processes occasionally fail, leading to alterations in the somatic genome, which has been recognized as a feature of both aging and cancer (Hoeijmakers, 2009; Hanahan & Weinberg, 2011; Vijg & Suh, 2013). While there is evidence for increased DNA repair activities in cells from longer-lived species (Hart & Setlow, 1974), and GM genes have been associated with the evolution of longevity (Jobson *et al*., 2010; Li & de Magalhaes, 2013), a systematic analysis of GM genes in species with greatly different lifespans is thus far lacking. Here, we present an analysis of GM genes in the NMR, an exceptionally long-lived rodent species in which no cases of cancer have been observed, vis-à-vis human and mouse, two species with starkly different lifespans, but a higher cancer risk at the end of life.

The NMR is the longest lived rodent known, with a maximum lifespan of 32 years—almost ten times longer than the mouse (Gorbunova, 2007). For at least 80% of their lives, NMRs show little signs of senescence, no age-related increase in mortality, and high fecundity (Buffenstein, 2008). In addition to such attenuated aging phenotypes, the NMR is also unusual for its pronounced cancer resistance (Liang *et al*., 2010), which, in part, has been explained by high molecular mass hyaluronan (Tian *et al*., 2013). We hypothesized that genetic differences in GM could explain the NMR's exceptional longevity and part of its cancer resistance. Hence, we performed a comparative analysis of GM genes in the NMR, mouse, and human genomes.

First, we compiled a list of GM genes, incorporating published gene lists (Ronen & Glickman, 2001; Han *et al*., 2013). The genes in our list are involved in a wide range of pathways and processes related to GM, from DNA repair to cell cycle regulation and cell death. Relatively well-annotated genome assemblies of human and mouse are available. For NMR, we used our published NMR genome assembly (Kim *et al*., 2011), as well as assemblies developed by several other groups (Table S2). As each genome assembly is independent, they complement one another—copy number variations can be validated and sequence gaps filled. We identified GM genes in the three species through genome mapping, refined local sequence alignment, and extensive manual checking (see Appendix S1).

While we found evidence of gene expansion and many putative pseudogenes, there were only two genes, CCAAT/enhancer binding protein-γ (*CEBPG*), and TERF1-interacting nuclear factor 2 (*TINF2*), with higher copy number in the NMR (Table1, Fig. S4). CEBPG has been identified as a regulator of DNA repair (Crawford *et al*., 2007) and cellular senescence (Huggins *et al*., 2013). Hence, an increased copy number of *CEBPG* may serve to better protect the NMR against cellular stressors. TINF2 stabilizes the shelterin proteins that prevent telomere uncapping and DNA damage signaling (Takai *et al*., 2011). Expression of two copies of *CEBPG* and both copies of *TINF2* in the NMR was verified with published RNA-seq data (Figs S5 and S6). The human genome contains only one copy of TINF2, but being large animals, humans are protected against cancer by repressing telomerase (Seluanov *et al*., 2007). One gene, present in the NMR and human genomes, but not in that of the mouse, encodes replication protein A4 (RPA4) (Table1, Fig. S4). RPA4 is a subunit of the replication protein A complex, which is essential for DNA replication, repair, and cell cycle checkpoint activation (Haring *et al*., 2010). While no orthologous sequences were found in the mouse genome, we identified an ortholog of the human *RPA4* sequence in the NMR genome (Fig. S4).

**Table 1:** Genome maintenance genes with differential copy numbers between human, mouse, and naked mole rat

Gene symbol	Copy numbers in					
	Human	Chimpanzee	Mouse	Rat	Guinea pig	NMR
CEBPG	1	1	1	1	1	3
GTF2H2C	2*	1	1	1	1	1
RPA4	1	1	0	0	0†	1
TINF2	1	1	1	1	2	2

* NCBI notes in the annotation of the second human copy of this gene that it may be an artifact of the Hg19 human genome assembly and may not actually be a true second copy of the gene (http://www.ncbi.nlm.nih.gov/gene/730394). However, this second copy is still in Ensembl, Refseq, and HGNC.

† Guinea pig has one partial copy of the RPA4 gene (Fig. S4).

While limitations in genome assemblies of most other species essentially constrained complete analysis, we did check the genomes of the guinea pig, chimpanzee, and rat for copy number variation in the four GM genes identified with copy number variation between human, mouse, and NMR. The results show that they each have only one copy of CEBPG, but like NMR, the guinea pig also has two copies of TINF2 (Table1). Guinea pigs also contain a partial sequence of *RPA4* in their genome, which was absent not only from the mouse but also from the rat genome (Table1). Finally, the human genome was found to contain an extra copy of general transcription factor IIH, polypeptide 2 (GTF2H2C), which is involved in basal transcription and nucleotide excision repair (Marteijn *et al*., 2014). However, this second copy may be an artifact of the Hg19 human genome assembly.

**Figure 1:**
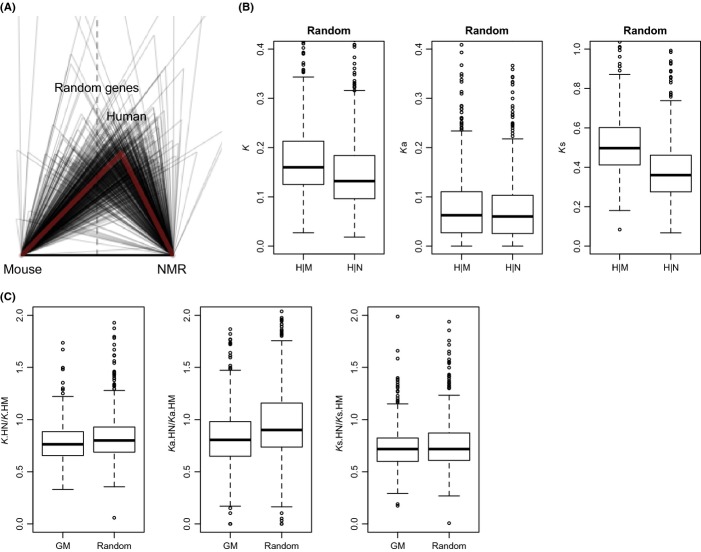
Evolution of genome maintenance (GM) and random genes in human, mouse, and naked mole rat (NMR). (A) Nucleotide substitutions per site. (B) Nucleotide substitutions per site (*K*), per nonsynonymous site (*K*a), and per synonymous site (*K*s). (C) The ratios of *K*,*K*a, and *K*s of GM and random genes in NMR to that in mouse. Abbreviations: H, human; M, mouse; and N, NMR.

The main conclusion that can be drawn from these results is that GM genes are highly conserved, also with respect to their copy numbers. If GM is superior in NMR (and human) compared with mouse, then we would expect to find that reflected by their germline mutation rates. We found that the number of nucleotide substitutions per site (*K*) in GM genes is on average 1.3 times higher between human and mouse than between human and NMR (Fig. 1A and Fig. S1A). Nucleotide substitution rates are known to be higher in rodents than in primates (Britten, 1986). Using chicken as an out-group, our calculation of *K* in ∽700 randomly selected genes confirmed this (Fig. S3). To investigate how nucleotide substitutions are distributed among different codon sites, we also calculated the nucleotide substitutions per nonsynonymous site (*K*a) and per synonymous site (*K*s) for human versus mouse and human versus NMR. Our estimated median *K*a/*K*s ratio between human and mouse orthologous genes is 0.119, in reasonable agreement with previous estimates (0.115) (Mouse Genome Sequencing Consortium *et al*., 2002). While *K*a is essentially the same in mouse and NMR, *K*s is higher in mouse than in NMR (*P *<* *2.2 × 10^−16^, for both GM and random genes), which indicates a lower background evolutionary rate in NMR than in mouse (Fig.1B and Fig. S1B). For neutral nucleotide substitutions, the substitution rate is equal to the mutation rate. As *K*s is lower in NMR than in mouse, the mutation rate is, therefore, also lower in NMR. Using human as an out-group, the comparison of *K*s among mouse, NMR, and guinea pig—a moderately long-lived rodent (with a 12-year maximum lifespan), shows that the maximum lifespan decreases as *K*s (and thus the mutation rate) increases (ordinal logistic regression coefficient = −2.12, *P *=* *2.31 × 10^−9^, Fig. S2). While species-specific differences in germline mutation rate have been attributed to various factors, varying from generation time to metabolic rate, the most likely explanation remains differences in genome maintenance (Thomas & Hahn, 2014). Moreover, nonsynonymous changes between human and mouse GM genes are slightly more drastic than those between human and NMR (*P *=* *0.03888), while for random genes such a difference was not observed (*P *=* *0.7555).

To investigate how a slower mutation rate affects GM genes in NMR, we calculated the ratios of *K* of GM genes between human and NMR to that between human and mouse, and compared them to those of random genes. The results indicate lower ratios of *K* for GM genes than for random genes (*P *=* *0.001, Fig.1C). Considering nonsynonymous and synonymous substitutions separately, we show that the aforementioned lower ratios result only from changes in nonsynonymous nucleotide substitutions, as the ratios of *K*a are significantly lower (*P *=* *2.248 × 10^−6^, Fig.1C) for GM genes while the ratios of *K*s are essentially the same between GM genes and random genes (*P *=* *0.2097, Fig.1C). This result indicates that GM genes evolved more slowly than random genes (the background) in NMR compared with mouse, and this reduction is due to a greater decrease in nonsynonymous nucleotide substitutions in GM genes than in random genes in NMR (or a greater increase in mouse). This result suggests that GM genes are evolutionarily more stable in NMR than in mouse, which may be required for longevity and/or resistance to cancer in NMR. Pathway analysis shows that the GM genes with the smallest *K*a ratios between NMR and human are enriched in the ubiquitin-mediated proteolysis pathway (*P*_*adj*_* *= 6.2 × 10^−15^), consistent with the notion that this pathway is extremely well conserved.

Our study is the first step in a comparative genomics approach to study GM in relation to aging and cancer. Focusing on human, mouse, and NMR because of their contrasting aging phenotypes and the availability of high-quality genome sequences, we investigated copy number differences of GM genes and discovered that very few GM genes have been lost among these three species during evolution. While we can only speculate whether the two genes with additional copies in the NMR, *CEBPG* and *TINF2*, confer a significant advantage, for example, through an increase in gene dosage, it is possible for a subtle difference at the genomic level to have a large phenotypic effect, such as increased lifespan. The finding that the NMR has a slower nucleotide substitution rate is interesting, particularly in the context of their longevity, and suggests that GM in NMR is superior to GM in the mouse. As more genomes become sequenced and annotated to higher quality, these findings can be validated further, elucidating the role of genome maintenance in modulating lifespan. Our findings in this comparative analysis of GM in human, mouse, and NMR suggest that NMR has more robust GM than mouse, which could play a role in the former's extreme longevity.

## Supporting Information

**Appendix S1** Methods. **Fig. S1** Genome maintenance (GM) genes in human, mouse, and NMR. **Fig. S2** Evolution of genome maintenance genes between human and mouse, naked mole-rat, and guinea pig, respectively. **Fig. S3** Evolution of random genes between chicken and human, mouse, and naked mole-rat, respectively. **Fig. S4** Sequence alignments and phylogenetic trees of genes with higher copy number in naked mole-rat than human or mouse. **Fig. S5** Alignment of RNA-seq reads to copies of CEBPG gene in NMR shows expression in two copies and none in the third copy. **Fig. S6** Alignment of RNA-seq reads to copies of TINF2 gene in NMR shows expression in both copies. **Table S1** Human genome maintenance genes. **Table S2** NMR genome sequencing and assemblies.

## References

[b1] Britten RJ (1986). Rates of DNA sequence evolution differ between taxonomic groups. Science.

[b2] Buffenstein R (2008). Negligible senescence in the longest living rodent, the naked mole-rat: insights from a successfully aging species. J. Comp. Physiol. B.

[b4] Crawford EL, Blomquist T, Mullins DN, Yoon Y, Hernandez DR, Al-Bagdhadi M, Ruiz J, Hammersley J, Willey JC (2007). CEBPG regulates ERCC5/XPG expression in human bronchial epithelial cells and this regulation is modified by E2F1/YY1 interactions. Carcinogenesis.

[b100] Gorbunova V, Seluanov A, Zhang ZD, Gladyshev VN, Vijg J (2014). Comparative genetics of longevity and cancer: insights from long-lived rodents. Nature Reviews Genetics.

[b5] Han J, Ryu S, Moskowitz DM, Rothenberg D, Leahy DJ, Atzmon G, Barzilai N, Suh Y (2013). Discovery of novel non-synonymous SNP variants in 988 candidate genes from 6 centenarians by target capture and next-generation sequencing. Mech. Ageing Dev.

[b6] Hanahan D, Weinberg RA (2011). Hallmarks of cancer: the next generation. Cell.

[b7] Haring SJ, Humphreys TD, Wold MS (2010). A naturally occurring human RPA subunit homolog does not support DNA replication or cell-cycle progression. Nucleic Acids Res.

[b8] Hart RW, Setlow RB (1974). Correlation between deoxyribonucleic acid excision-repair and life-span in a number of mammalian species. Proc. Natl. Acad. Sci. U.S.A.

[b9] Hoeijmakers JH (2009). DNA damage, aging, and cancer. N. Engl. J. Med.

[b10] Huggins CJ, Malik R, Lee S, Salotti J, Thomas S, Martin N, Quinones OA, Alvord WG, Olanich ME, Keller JR, Johnson PF (2013). C/EBPgamma suppresses senescence and inflammatory gene expression by heterodimerizing with C/EBPbeta. Mol. Cell. Biol.

[b11] Jobson RW, Nabholz B, Galtier N (2010). An evolutionary genome scan for longevity-related natural selection in mammals. Mol. Biol. Evol.

[b12] Kim EB, Fang X, Fushan AA, Huang Z, Lobanov AV, Han L, Marino SM, Sun X, Turanov AA, Yang P, Yim SH, Zhao X, Kasaikina MV, Stoletzki N, Peng C, Polak P, Xiong Z, Kiezun A, Zhu Y, Chen Y, Kryukov GV, Zhang Q, Peshkin L, Yang L, Bronson RT, Buffenstein R, Wang B, Han C, Li Q, Chen L, Zhao W, Sunyaev SR, Park TJ, Zhang G, Wang J, Gladyshev VN (2011). Genome sequencing reveals insights into physiology and longevity of the naked mole rat. Nature.

[b13] Li Y, de Magalhaes JP (2013). Accelerated protein evolution analysis reveals genes and pathways associated with the evolution of mammalian longevity. Age.

[b14] Liang S, Mele J, Wu Y, Buffenstein R, Hornsby PJ (2010). Resistance to experimental tumorigenesis in cells of a long-lived mammal, the naked mole-rat (*Heterocephalus glaber*. Aging Cell.

[b103] Marteijn JA, Lans H, Vermeulen W, Hoeijmakers JH (2014). Understanding nucleotide excision repair and its roles in cancer and ageing. Nat Rev Mol Cell Biol.

[b15] Waterston RH, Lindblad-Toh K, Birney E, Rogers J, Abril JF, Agarwal P, Agarwala R, Ainscough R, Alexandersson M, An P, Antonarakis SE, Attwood J, Baertsch R, Bailey J, Barlow K, Beck S, Berry E, Birren B, Bloom T, Bork P, Botcherby M, Bray N, Brent MR, Brown DG, Brown SD, Bult C, Burton J, Butler J, Campbell RD, Carninci P, Cawley S, Chiaromonte F, Chinwalla AT, Church DM, Clamp M, Clee C, Collins FS, Cook LL, Copley RR, Coulson A, Couronne O, Cuff J, Curwen V, Cutts T, Daly M, David R, Davies J, Delehaunty KD, Deri J, Dermitzakis ET, Dewey C, Dickens NJ, Diekhans M, Dodge S, Dubchak I, Dunn DM, Eddy SR, Elnitski L, Emes RD, Eswara P, Eyras E, Felsenfeld A, Fewell GA, Flicek P, Foley K, Frankel WN, Fulton LA, Fulton RS, Furey TS, Gage D, Gibbs RA, Glusman G, Gnerre S, Goldman N, Goodstadt L, Grafham D, Graves TA, Green ED, Gregory S, Guigo R, Guyer M, Hardison RC, Haussler D, Hayashizaki Y, Hillier LW, Hinrichs A, Hlavina W, Holzer T, Hsu F, Hua A, Hubbard T, Hunt A, Jackson I, Jaffe DB, Johnson LS, Jones M, Jones TA, Joy A, Kamal M, Karlsson EK, Karolchik D, Kasprzyk A, Kawai J, Keibler E, Kells C, Kent WJ, Kirby A, Kolbe DL, Korf I, Kucherlapati RS, Kulbokas EJ, Kulp D, Landers T, Leger JP, Leonard S, Letunic I, Levine R, Li J, Li M, Lloyd C, Lucas S, Ma B, Maglott DR, Mardis ER, Matthews L, Mauceli E, Mayer JH, McCarthy M, McCombie WR, McLaren S, McLay K, McPherson JD, Meldrim J, Meredith B, Mesirov JP, Miller W, Miner TL, Mongin E, Montgomery KT, Morgan M, Mott R, Mullikin JC, Muzny DM, Nash WE, Nelson JO, Nhan MN, Nicol R, Ning Z, Nusbaum C, O’ Connor MJ, Okazaki Y, Oliver K, Overton-Larty E, Pachter L, Parra G, Pepin KH, Peterson J, Pevzner P, Plumb R, Pohl CS, Poliakov A, Ponce TC, Ponting CP, Potter S, Quail M, Reymond A, Roe BA, Roskin KM, Rubin EM, Rust AG, Santos R, Sapojnikov V, Schultz B, Schultz J, Schwartz MS, Schwartz S, Scott C, Seaman S, Searle S, Sharpe T, Sheridan A, Shownkeen R, Sims S, Singer JB, Slater G, Smit A, Smith DR, Spencer B, Stabenau A, Stange-Thomann N, Sugnet C, Suyama M, Tesler G, Thompson J, Torrents D, Trevaskis E, Tromp J, Ucla C, Ureta-Vidal A, Vinson JP, Von Niederhausern AC, Wade CM, Wall M, Weber RJ, Weiss RB, Wendl MC, West AP, Wetterstrand K, Wheeler R, Whelan S, Wierzbowski J, Willey D, Williams S, Wilson RK, Winter E, Worley KC, Wyman D, Yang S, Yang SP, Zdobnov EM, Zody MC, Lander ES, Mouse Genome Sequencing Consortium (2002). Initial sequencing and comparative analysis of the mouse genome. Nature.

[b16] Ronen A, Glickman BW (2001). Human DNA repair genes. Environ. Mol. Mutagen.

[b102] Seluanov A, Chen ZX, Hine C, Sasahara THC, Ribeiro AACM, Catania KC, Presgraves DC, Gorbunova V (2007). Telomerase activity coevolves with body mass not lifespan. Aging Cell.

[b17] Takai K, Kibe T, Donigian Jill R, Frescas D, de Lange T (2011). Telomere protection by TPP1/POT1 requires tethering to TIN2. Mol. Cell.

[b18] Thomas GWC, Hahn MW (2014). The human mutation rate is increasing, even as it slows. Mol. Biol. Evol.

[b19] Tian X, Azpurua J, Hine C, Vaidya A, Myakishev-Rempel M, Ablaeva J, Mao Z, Nevo E, Gorbunova V, Seluanov A (2013). High-molecular-mass hyaluronan mediates the cancer resistance of the naked mole rat. Nature.

[b20] Vijg J, Suh Y (2013). Genome instability and aging. Annu. Rev. Physiol.

