# Supplementary material for: Comparative analysis of genome maintenance genes in naked mole rat, mouse, and human
Source: Aging Cell. 2015 Jan 28;14(2):288–91. doi: 10.1111/acel.12314 (PMC4364841; doi:10.1111/acel.12314)
Supplement: Supplementary file 1 [file acel0014-0288-sd1.pdf]

Supporting Information

METHODS

The genomic sequences used in this study were human genome build hg19 (February 2009), mouse genome build mm9 (July 2007), three naked mole rat (NMR) genome assemblies (Supplementary Table 2), guinea pig genome build cavPor3 (February 2008), chicken genome build galGal4 (November 2011), rat genome build Rn5 (March 2012), and chimpanzee genome build PanTro4 (February 2011). The Broad assembly of the naked mole rat genome, as well as our own assembly, was checked when verifying gene copy numbers for genome maintenance genes. Given a set of human genes (e.g., genome maintenance genes or ~700 randomly selected genes), we used Homologene (http://www.ncbi.nlm.nih.gov/homologene) and InParanoid (Ostlund et al., 2010) to identify their mouse and chicken orthologues. For each gene, we obtained the NCBI RefSeq protein sequences of all its isoforms (http://www.ncbi.nlm.nih.gov/refseq/).

Identifying genome maintenance genes in the naked mole rat genome

Using BLAT (Kent, 2002), we mapped human and mouse genome maintenance protein sequences to their corresponding genome sequences. To identify naked mole rat orthologs, we mapped the mouse protein sequences to naked mole rat genome sequences. If the mouse protein sequences were missing, we used the human protein sequences instead. For each BLAT mapping of a protein sequence to a genome, we only kept the best alignment and any alignments whose scores are within 2/3 of the score of the best alignment. We extracted the sequences at the genomic locations of the filtered BLAT alignments and then used GeneWise (Birney et al., 2004) to obtain finer and more detailed alignments between the protein sequences and the extracted genomic nucleotide sequences. To reduce false positives, we manually checked the validity of each GeneWise alignment.

Validating genes in NMR genome

Genes with multiple copies in NMR, along with the RPA4 gene, were validated by aligning raw genome sequencing reads to the target sequences using the Burrows-Wheeler aligner (http://bio-bwa.sourceforge.net/; Li and Durbin, 2009). Multiple sequence alignments and phylogenetic tress were generated using Geneious (http://www.geneious.com; Kearse et al., 2012). Expression of these genes was confirmed using RNA-seq reads from the Gladyshev laboratory (Fang et al., 2014). Raw RNA-seq data files (FASTQ) from the naked mole rat brain and liver were converted to FASTA using custom Perl and bash scripts. The multiple sequence aligner MUSCLE was used to identify unique regions of putative duplicated gene sequences/paralogues. Unique regions of paralogues were used to query RNA-seq BLAST databases on a local instance of the Ruby-based SequenceServer (http://www.sequenceserver.com).

Validating gene copy numbers in other mammalian genomes

We validated the copy numbers of genes in Table 1 in the human, chimpanzee, mouse, rat, guinea pig, and NMR genomes using BLAT and the UCSC genome browser

(http://genome.ucsc.edu/; Kent et al., 2002) to map the corresponding human and mouse protein sequences to the different genomes, looking for full length sequences, and used GeneWise and MUSCLE alignments to check for pseudogenes , gene structure, and homology.

We also checked whether the two genome maintenance genes duplicated in the NMR genome, TINF2 and CEBGP, are also duplicated in the closely related African mole rat species, the Damaraland mole rat (DMR) (Fang et al., 2014). We found only one copy each of TINF2 and CEBGP in the DMR genome using the GLEAN gene annotation pipeline and GMAP (Wu and Watanabe, 2005) Thus, as the NMR and DMR separated ~26 million years ago, the genome maintenance duplications we found may contribute to the 10+ years longer lifespan of the NMR.

Comparing nucleotide substitution rates

To study how genes evolved in human, mouse, and naked mole rat, for each gene, we first extracted from GeneWise alignment files for the longest coding and peptide sequences from each species. We then aligned the nucleotide coding sequences (based on the alignment of their corresponding peptide sequences) and calculated the number of nucleotide substitutions per site between each possible pair of the three species. We aligned the coding sequences based on the alignment of their corresponding peptide sequences that we generated using MUSCLE (Edgar, 2004). For each GM or random gene, we used Kimura's 2-parameters model (Kimura, 1980) to calculate K^{HM}, K^{HN}, and K^{MN}, the nucleotide substitutions per site for the human-mouse, human-NMR, and mouse-NMR sequence comparisons, respectively. For these protein-coding genes, we also calculated *K*a and *K*s, the number of substitutions per synonymous site and per nonsynonymous site, for all three comparisons using Li's method (Li, 1993). To calculate the substitution rates, we used the divergence time reported previously (Kim et al., 2011): 93 million year ago (MYA) between human and rodent and 73 MYA between mouse and naked mole rat.

To compare *K*, *K*a, and *K*s of a GM gene in each of mouse, naked mole rat, and guinea pig lineages separately, we used the coding sequence of its human ortholog as the out-group and calculated *K*, *K*a, and *K*s between human and mouse, naked mole rat, and guinea pig respectively (Supplementary Figure 2).

To compare *K*, *K*a, and *K*s of a radom gene in each of human, mouse, and naked mole rat lineages separately, we also used the coding sequence of its chicken ortholog as the out-group and calculated *K*, *K*a, and *K*s between chicken and human, mouse, and naked mole rat, respectively (Supplementary Figure 3).

We also investigated the nature of nonsynonymous codon changes to the protein coding sequences among human, mouse, and naked mole rat. To do this, we used the aforementioned MUSCLE-generated peptide sequence alignments. Given the alignment of a particular gene, we only considered the residue sites with at least one sequence change, ignoring gaps and invariant sites among all three species. Using BLOSUM62, we scored the pairwise comparison between two species at these sites with nonsynonymous substitutions in a gene, and then summed these scores to give a final score for the gene between the two species. The smaller the score, the more different the two sequences are (and thus more deleterious the changes are). We calculated the scores of the human-mouse, human-NMR, and mouse-NMR sequence comparisons for all genome maintenance genes and a set of random genes.

By using the human gene sequences as the reference, it can be seen that the alignment scores of genome maintenance genes are higher for naked mole rat than mouse (P = 0.03888 by Wilcoxon rank sum test). However, randomly selected genes do not show this tendency (P = 0.7555 by Wilcoxon rank sum test). The results indicate that genome maintenance genes are more conserved in naked mole rat than that in mouse.

We used DAVID (http://david.abcc.ncifcrf.gov/) to analyze pathway enrichment among GM genes with the smallest *K*a ratios between NMR and human.

SUPPLEMENTARY TABLES

Supplementary Table 1. Human genome maintenance genes

AATF ABL1 ACD AICDA AIRE ALKBH1 ALKBH2 ALKBH3 ANAPC10 ANAPC11 ANAPC13 ANAPC2 ANAPC4 ANAPC5 ANAPC7 APEX1 APEX2 APITD1 APLF APTX ARHGEF7 ASF1A ASTE1 ATG12 ATG5 ATM ATMIN ATR ATRIP ATRX ATXN8 AXIN2 BABAM1 BARD1 BAZ1B BCCIP BHLHE40 BIRC6 BLM BRCA1 BRCA2 BRCC3 BRE BRIP1 BTG2 BTRC BUB1 C11ORF30 C15ORF42 C17ORF28 C17ORF70 C19ORF40 C1ORF124 C9ORF102 C9ORF80 CBL CCNH CCNO CD247 CDC14B CDC16 CDC20 CDC23 CDC25A CDC25B CDC25C CDC26 CDC27 CDCA5 CDK7 CDKN2A CDKN2D CEBPG CEP164 CETN2 CHAF1A CHAF1B CHD1L CHEK1 CHEK2 CHRNA4 CIB1 CINP CLSPN CREBBP CRY1 CRY2 CSNK1D CSNK1E CSNK2A1 CSNK2A2 CSNK2B CUL1 CUL2 CUL3 CUL4A CUL4B CUL5 CUL7 DCLRE1A DCLRE1B DCLRE1C DDB1 DDB2 DDX1 DEPDC1B DET1 DNA2 DNASE1 DTL DUSP1 E2F1 EEPD1 EME1 EME2 EP300 EPC2 ERCC1 ERCC2 ERCC3 ERCC4 ERCC5 ERCC6 ERCC8 ESCO1 ESCO2 EXO1 EYA1 EYA2 EYA3 EYA4 FAM175A FANCA FANCC FANCD2 FANCE FANCF FANCG FANCI FANCL FANCM FBXO18 FBXO2 FBXO4 FBXO6 FBXW7 FBXW8 FEN1 FGF10 FSBP FSCN1 FTO FZR1 GABARAPL1 GADD45A GADD45B GADD45G GEN1 GTF2H1 GTF2H2 GTF2H2C GTF2H3 GTF2H4 GTF2H5 H2AFX HDAC9 HERC1 HERC2 HERC3 HERC4 HINFP HLTF HMGB1 HMGB1P10 HMGB2 HUS1 HUWE1 IGHMBP2 IL12B INTS3 ITCH JMY KAT5 KIAA1530 KIAA2022 KIF22 KIN LIG4 LMNA LRIG1 LRIG3 MAD2L2 MAP3K1 MBD4 MC1R MDC1 MDM2 MEN1 MGMT MGRN1 MLH1 MLH3 MMS19 MMS22L MNAT1 MORF4L1 MORF4L2 MPG MRE11A MRPL36 MSH2 MSH3 MSH4 MSH5 MSH6 MUM1 MUS81 MUTYH MYC NBN NCOA6 NEDD4 NEIL1 NEIL2 NEIL3 NF1 NHEJ1 NHLRC1 NOD2 NONO NPM1 NR1H2 NSMCE1 NSMCE2 NTHL1 NUDT1 OBFC2A OBFC2B OGG1 PALB2 PAPD7 PARG PARP1 PARP2 PARP3 PARP4 PAXIP1 PCNA PDLIM3 PIAS1 PIAS4 PINX1 PIPSL PML PMS1 PMS2 PMS2L2 PMS2P1 PMS2P12 PMS2P5 PNKP POLA1 POLB POLD1 POLD2 POLD3 POLDIP2 POLE POLE2 POLE3 POLE4 POLG POLG2 POLI POLL POLM POLN POLQ POLR1A POLR1B POLR2A POLR2B POLR2C POLR2D POLR2E POLR2F POLR2G POLR2H POLR2I POLR2J POLR2K POLR2L POT1 POU1F1 POU2F1 POU2F2 POU2F3 POU3F1 POU3F2 POU3F3 POU3F4 POU4F1 POU4F2 POU4F3 POU5F1 POU5F2 POU6F1 POU6F2 PPIL2 PRDX2 PRKCG PRKDC PRMT6 PROC PRPF19 PTEN PTTG1 RAD1 RAD17 RAD18 RAD21 RAD23A RAD23B RAD50 RAD51 RAD51AP1 RAD51B RAD51C RAD51D RAD52 RAD54B RAD54L RAD9A RAD9B RAG1 RBBP8 RBM14 RBX1 RCHY1 RDM1 RDX RECQL RECQL4 RECQL5 REV1 REV3L RFC1 RFC2 RFC3 RFC4 RFC5 RFWD2 RFWD3 RHOBTB2 RNF168 RNF169 RNF4 RNF8 RPA3 RPA4 RPAIN RPS27L RPS3 RRM2B RTEL1 RUVBL2 SAE1 SCARA3 SENP1 SENP2 SENP3 SENP5 SENP6 SENP7 SENP8 SETMAR SETX SFPQ SHFM1 SHPRH SIAH1 SIRT1 SIRT2 SIRT3 SIRT4 SIRT5 SIRT6 SIRT7 SKP1 SKP2 SLC25A24 SLC30A9 SLK SLX1B SLX4 SMC1A SMC3 SMC5 SMC6 SMG1 SMUG1 SOCS1 SOCS3 SOD1 SRBD1 SSRP1 STAG2 STRAP STUB1 SUMO1 SUMO1P1 SUMO1P3 SUMO2 SUMO4 SUPT16H SUPT6H SYVN1 TBP TCEA1 TCEB1 TCEB2 TDG TDP1 TDP2 TERF1 TERF2 TERF2IP TERT TGFB1 TINF2 TMEM161A TNKS TNP1 TONSL TOP2A TOPBP1 TP53 TP53BP1 TP73 TRAF6 TRANK1 TREX1 TREX2 TRIM31 TRIM32 TRIM37 TRIP12 TRIP13 TTC5 TXN TYMS UBA1 UBA2 UBA3 UBA52 UBB UBE2A UBE2B UBE2C UBE2D2 UBE2D3 UBE2F UBE2G1 UBE2G2 UBE2H UBE2I UBE2J1 UBE2J2 UBE2K UBE2L3 UBE2L6 UBE2M UBE2N UBE2O UBE2Q1 UBE2S UBE2T UBE2U UBE2V1 UBE2V2 UBE2W UBE2Z UBE3B UBE3C UBE4A UBE4B UBOX5 UBR5 UHRF1 UIMC1 UNG UPF1 URM1 USP1 USP10 USP28 USP3 UVRAG VCP VHL WDR33 WRN WRNIP1 WWP1 WWP2 XAB2 XIRP1 XPA XPC XRCC1 XRCC2 XRCC3 XRCC4 XRCC5 XRCC6 XRCC6BP1 ZBTB32 ZSWIM7

Supplementary Table 2. NMR genome sequencing and assemblies.

NMR genome	Sequencing libraries	Assembly method	Depth	Contig N50 (kb)	Scaffold N50 (kb)
Albert Einstein College of Medicine	0.18 and 3 kb	ALLPATHS-LG	45x	16.7	62
BGI HetGla_1.0, Kim *et al.* 2011[1]	0.17, 0.35, 0.5, 0.8, 2, 5, 10, and 20 kb	SOAP-denovo	92x	19.3	1600
HetGla_female_1.0, Broad Institute[2]	0.18,3, 6-14, and 37.5 kb	ALLPATHS-LG	90x	47.8	20,532

Notes:
1. http://www.ncbi.nlm.nih.gov/assembly/310228 (Fang et al., 2014)
2. http://www.ncbi.nlm.nih.gov/assembly/362148/ (Keane et al., 2014)

SUPPLEMENTARY FIGURES

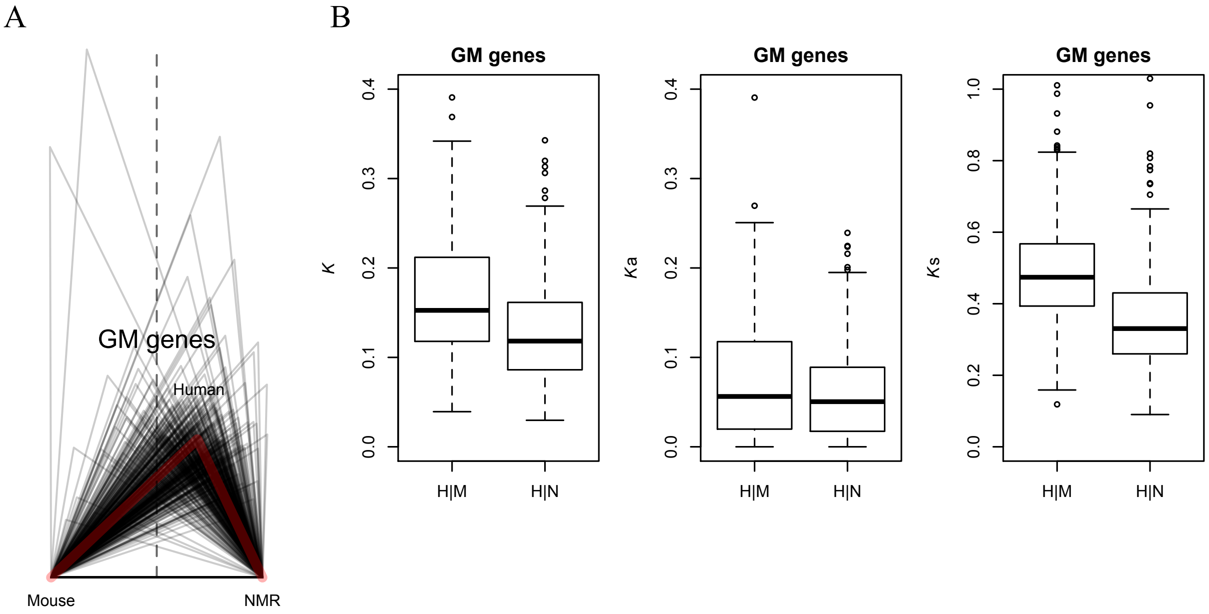

Supplementary Figure 1. Genome maintenance (GM) genes in human, mouse, and NMR. (A). Nucleotide substitutions per site (*K*). For each GM gene, a triangle is constructed with each side equal to *K* between a pair of species and then scaled so the M-NMR side becomes unity. The average scaled triangle is plotted in red. The dashed line denotes the top vertex of the isosceles, which represents genes with equal *K* between H-M and H-NMR. (B) Nucleotide substitutions per site (*K*), per non-synonymous site (*K*a), and per synonymous site (*K*s) of GM genes. Abbreviations: H, human; M, mouse; and N, NMR.

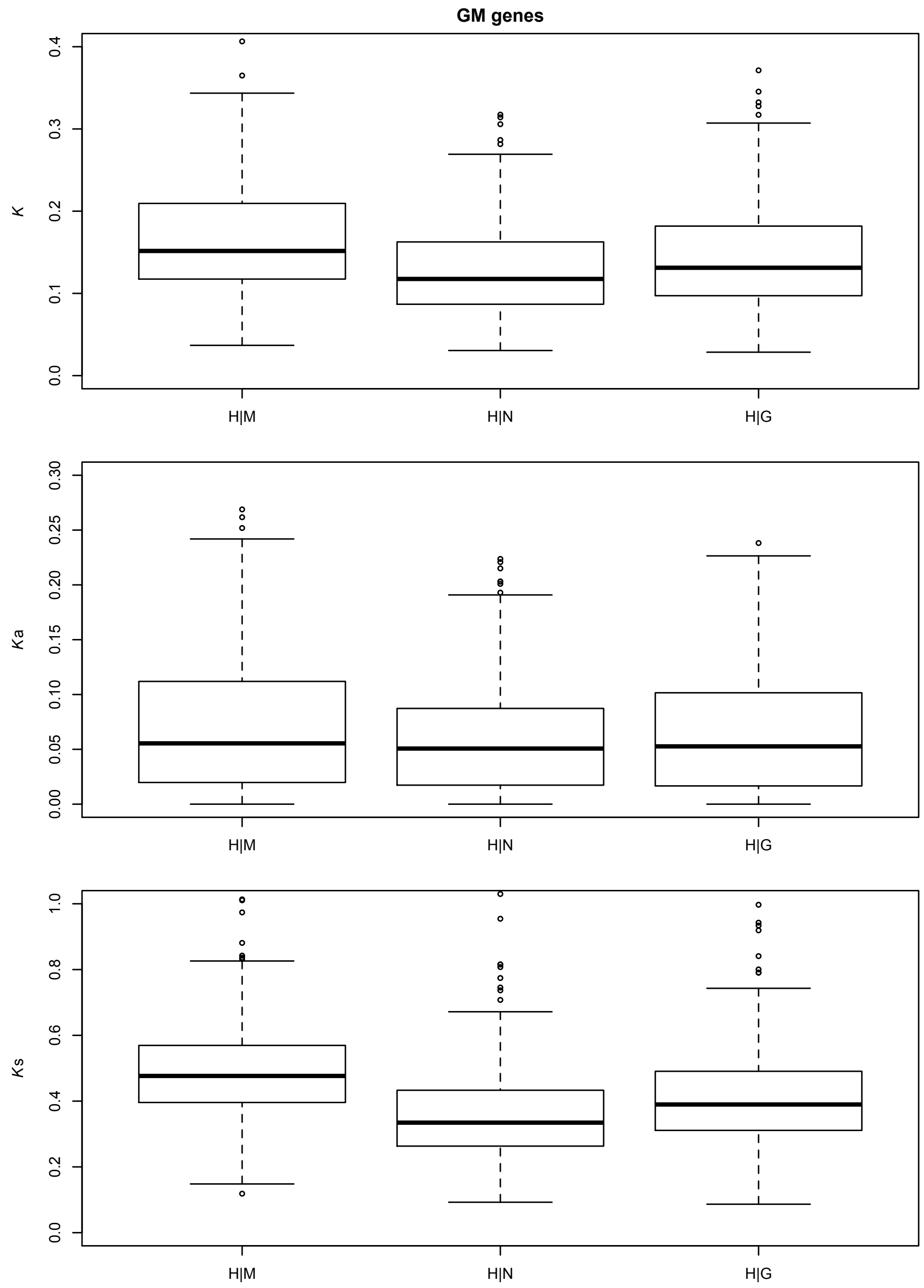

Supplementary Figure 2. Evolution of genome maintenance genes between human and mouse, naked mole rat, and guinea pig, respectively. Abbreviations: H, human; M, mouse; N, NMR; and G, guinea pig.

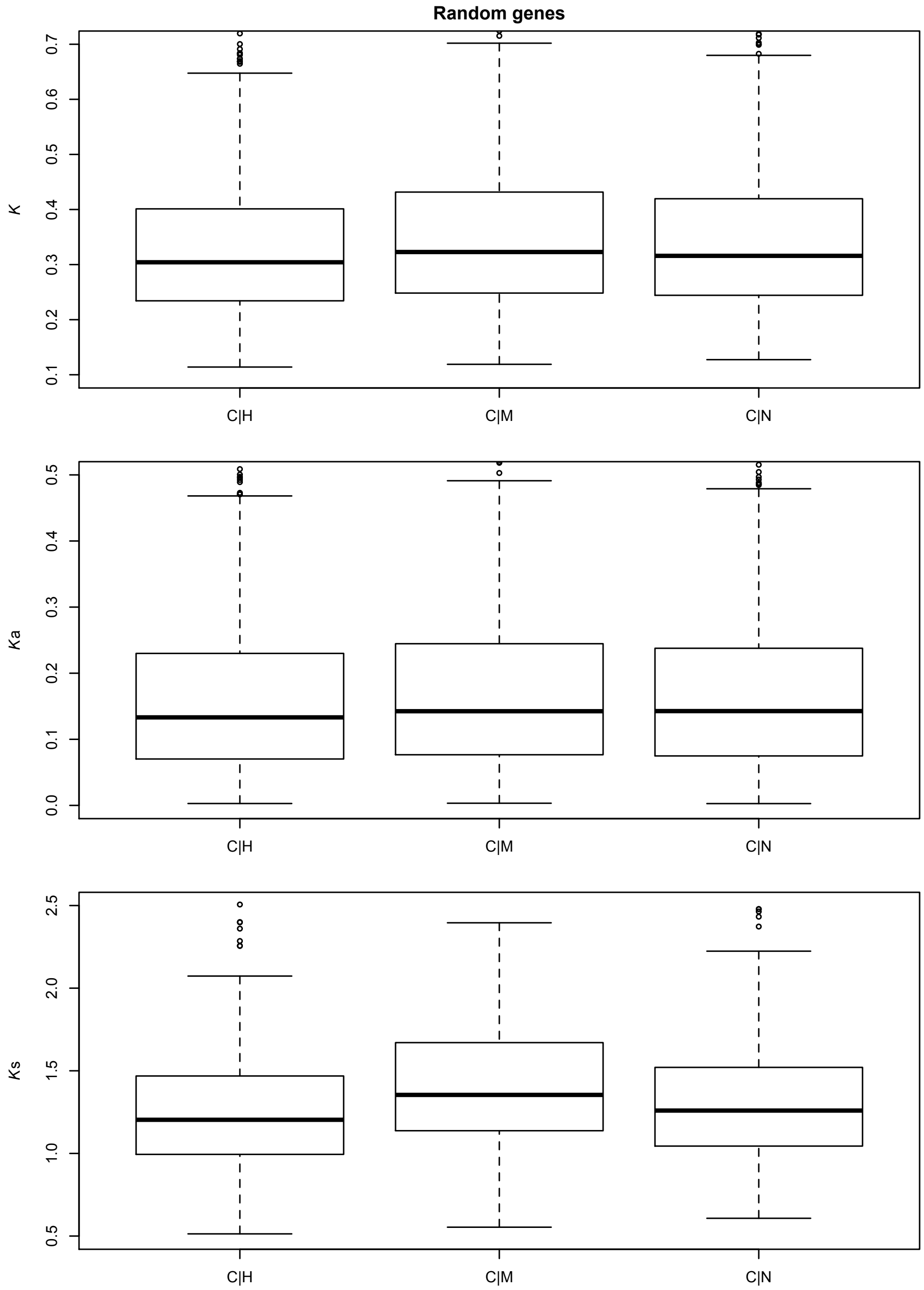

Supplementary Figure 3. Evolution of random genes between chicken and human, mouse, and naked mole rat, respectively. Abbreviations: C, chicken; H, human; M, mouse; and N, NMR.

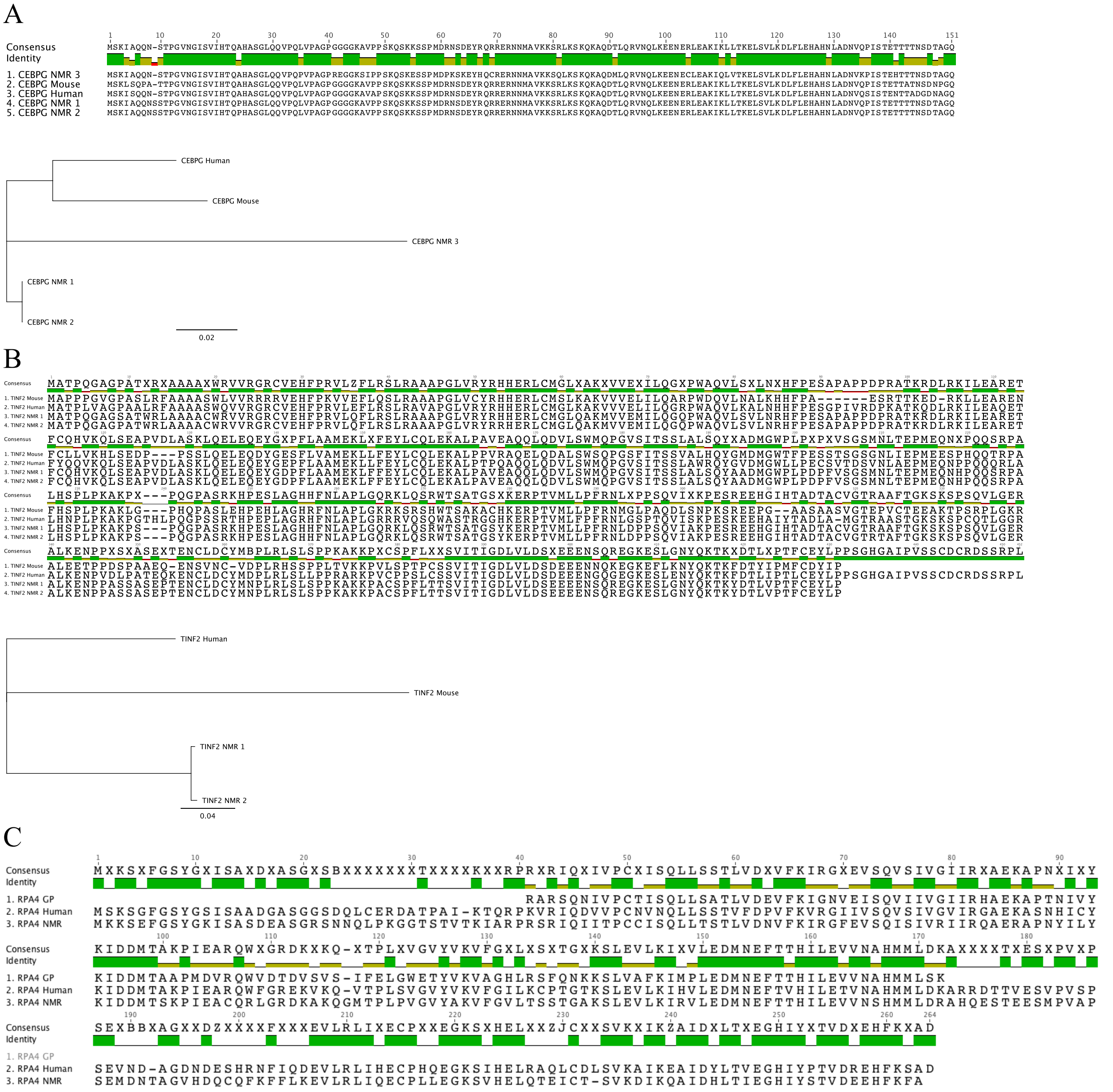

Supplementary Figure 4. Sequence alignments and phylogenetic trees of genes with higher copy number in naked mole rat than human or mouse. (A) CEBPG sequence alignment and tree for human, mouse, and NMR three copies (B) TINF2 sequence alignment and tree for human, mouse, and NMR two copies (C) RPA4 sequence alignment for human, NMR, and guinea pig (GP).

CEBPG

denotes evidence of transcription

```
CLUSTAL W (1.81) multiple sequence alignment

JH171653.1||746770-749193-CEBPG2      NNNNNNNNNNNNNNNNNNNNNNNNNNNNNNNNNNNNNNNNNNNNNNNNNNNNNNNNNNNN
JH169046.1||191678-194128-CEBPG-      ------------------------------------------------------------
JH172334.1||2060321-2062744-CEBP      ------------------------------------------------------------


JH171653.1||746770-749193-CEBPG2      NNNNNNNNNNNNNNNNNNNNNNNNNNNNNNNNNNNNNNNNNNNNNNNNNNNNNNNNNNNN
JH169046.1||191678-194128-CEBPG-      ------------------------------------------------------------
JH172334.1||2060321-2062744-CEBP      ------------------------------------------------------------


JH171653.1||746770-749193-CEBPG2      NNNNNNNNNNNNNNNNNNNNNNNNNNNNNNNNNNNNNNNNNNNNNNNNNNNNNNNNNNNN
JH169046.1||191678-194128-CEBPG-      ------------------------NNNNNNNNNNNNNNNNNNNNNNNNNNNNNNNNNNNN
JH172334.1||2060321-2062744-CEBP      -----------------------NNNNNNNNNNNNNNNNNNNNNNNNNNNNNNNNNNNNN
                                                              ************************************

JH171653.1||746770-749193-CEBPG2      NNNNNNNNNNNNNNNNNNNNNNNNNNNNNNNNNNNNNNNNNNNNNNNNNNNNNNNNNNNN
JH169046.1||191678-194128-CEBPG-      NNNNNNNNNNNNNNNNNNNNNNNNNNNNNNNNNNNNNNNNNNNNNNNNNNNNNNNNNNNN
JH172334.1||2060321-2062744-CEBP      NNNNNNNNNNNNNNNNNNNNNNNNNNNNNNNNNNNNNNNNNNNNNNNNNNNNNNNNNNNN
                                      ************************************************************

JH171653.1||746770-749193-CEBPG2      NNNNNNNNNNNNNNNNNNNNNNNNNNNNNNNNNNNNNNNNNNNNNNNNNNNNNNNNNNNN
JH169046.1||191678-194128-CEBPG-      NNNNNNNNNNNNNNNNNNNNNNNNNNNNNNNNNNNNNNNNNNNNNNNNNNNNNNNNNNNN
JH172334.1||2060321-2062744-CEBP      NNNNNNNNNNNNNNNNNNNNNNNNNNNNNNNNNNNNNNNNNNNNNNNNNNNNNNNNNNNN
                                      ************************************************************

JH171653.1||746770-749193-CEBPG2      NNNNNNNNNNNNNNNNNNNNNNNNNNNNNNNNNNNNNNNNNNNNNNNNNNNNNNNNNNNN
JH169046.1||191678-194128-CEBPG-      NNNNNNNNNNNNNNNNNNNNNNNNNNNNNNNNNNNNNNNNNNNNNNNNNNNNNNNNNNNN
JH172334.1||2060321-2062744-CEBP      NNNNNNNNNNNNNNNNNNNNNNNNNNNNNNNNNNNNNNNNNNNNNNNNNNNNNNNNNNNN
                                      ************************************************************

JH171653.1||746770-749193-CEBPG2      NNNNNNNNNNNNNNNNNNNNNNNNNNNNNNNNNNNNNNNNNNNNNNNNNNNNNNNNNNNN
JH169046.1||191678-194128-CEBPG-      NNNNNNNNNNNNNNNNNNNNNNNNNNNNNNNNNNNNNNNNNNNNNNNNNNNNNNNNNNNN
JH172334.1||2060321-2062744-CEBP      NNNNNNNNNNNNNNNNNNNNNNNNNNNNNNNNNNNNNNNNNNNNNNNNNNNNNNNNNNNN
                                      ************************************************************

JH171653.1||746770-749193-CEBPG2      NNNNNNNNNNNNNNNNNNNNNNNNNNNNNNNNNNNNNNNNNNNNNNNNNNNNNNNNNNNN
JH169046.1||191678-194128-CEBPG-      NNNNNNNNNNNNNNNNNNNNNNNNNNNNNNNNNNNNNNNNNNNNNNNNNNNNNNNNNNNN
JH172334.1||2060321-2062744-CEBP      NNNNNNNNNNNNNNNNNNNNNNNNNNNNNNNNNNNNNNNNNNNNNNNNNNNNNNNNNNNN
                                      ************************************************************

JH171653.1||746770-749193-CEBPG2      NNNNNNNNNNNNNNNNNNNNNNNNNNNNNNNNNNNNNNNNNNNNNNNNNNNNNNNNNNNN
JH169046.1||191678-194128-CEBPG-      NNNNNNNNNNNNNNNNNNNNNNNNNNNNNNNNNNNNNNNNNNNNNNNNNNNNNNNNNNNN
JH172334.1||2060321-2062744-CEBP      NNNNNNNNNNNNNNNNNNNNNNNNNNNNNNNNNNNNNNNNNNNNNNNNNNNNNNNNNNNN
                                      ************************************************************

JH171653.1||746770-749193-CEBPG2      NNNNNNNNNNNNNNNNNNNNNNNNNNNNNNNNNNNNNNNNNNNNNNNNNNNNNNNNNNNN
JH169046.1||191678-194128-CEBPG-      NNNNNNNNNNNNNNNNNNNNNNNNNNNNNNNNNNNNNNNNNNNNNNNNNNNNNNNNNNNN
JH172334.1||2060321-2062744-CEBP      NNNNNNNNNNNNNNNNNNNNNNNNNNNNNNNNNNNNNNNNNNNNNNNNNNNNNNNNNNNN
                                      ************************************************************

JH171653.1||746770-749193-CEBPG2      NNNNNNNNNNNNNNNNNNNNNNNNNNNNNNNNNNNNNNNNNNNNNNNNNNNNNNNNNNNN
JH169046.1||191678-194128-CEBPG-      NNNNNNNNNNNNNNNNNNNNNNNNNNNNNNNNNNNNNNNNNNNNNNNNNNNNNNNNNNNN
JH172334.1||2060321-2062744-CEBP      NNNNNNNNNNNNNNNNNNNNNNNNNNNNNNNNNNNNNNNNNNNNNNNNNNNNNNNNNNNN
                                      ************************************************************

JH171653.1||746770-749193-CEBPG2      NNNNNNNNNNNNNNNNNNNNNNNNNNNNNNNNNNNNNNNNNNNNNNNNNNNNNNNNNNNN
JH169046.1||191678-194128-CEBPG-      NNNNNNNNNNNNNNNNNNNNNNNNNNNNNNNNNNNNNNNNNNNNNNNNNNNNNNNNNNNN
JH172334.1||2060321-2062744-CEBP      NNNNNNNNNNNNNNNNNNNNNNNNNNNNNNNNNNNNNNNNNNNNNNNNNNNNNNNNNNNN
                                      ************************************************************

JH171653.1||746770-749193-CEBPG2      NNNNNNNNNNNNNNNNNNNNNNNNNNNNNNNNNNNNNNNNNNNNNNNNNNNNNNNNNNNN
JH169046.1||191678-194128-CEBPG-      NNNNNNNNNNNNNNNNNNNNNNNNNNNNNNNNNNNNNNNNNNNNNNNNNNNNNNNNNNNN
JH172334.1||2060321-2062744-CEBP      NNNNNNNNNNNNNNNNNNNNNNNNNNNNNNNNNNNNNNNNNNNNNNNNNNNNNNNNNNNN
                                      ************************************************************

JH171653.1||746770-749193-CEBPG2      NNNNNNNNNNNNNNNNNNNNNNNNNNNNNNNNNNNNNNNNNNNNNNNNNNNNNNNNNNNN
JH169046.1||191678-194128-CEBPG-      NNNNNNNNNNNNNNNNNNNNNNNNNNNNNNNNNNNNNNNNNNNNNNNNNNNNNNNNNNNN
JH172334.1||2060321-2062744-CEBP      NNNNNNNNNNNNNNNNNNNNNNNNNNNNNNNNNNNNNNNNNNNNNNNNNNNNNNNNNNNN
                                      ************************************************************

JH171653.1||746770-749193-CEBPG2      NNNNNNNNNNNNNNNNNNNNNNNNNNNNNNNNNNNNNNNNNNNNNNNNNNNNNNNNNNNN
```

```
JH169046.1||191678-194128-CEBPG-      NNNNNNNNNNNNNNNNNNNNNNNNNNNNNNNNNNNNNNNNNNNNNNNNNNNNNNNNNNNN
JH172334.1||2060321-2062744-CEBP      NNNNNNNNNNNNNNNNNNNNNNNNNNNNNNNNNNNNNNNNNNNNNNNNNNNNNNNNNNNN
                                      ************************************************************

JH171653.1||746770-749193-CEBPG2      NNNNNNNNNNNNNNNNNNNNNNNNNNNNNNNNNNNNNNNNNNNNNNNNNNNNNNNNNNNN
JH169046.1||191678-194128-CEBPG-      NNNNNNNNNNNNNNNNNNNNNNNNNNNNNNNNNNNNNNNNNNNNNNNNNNNNNNNNNNNN
JH172334.1||2060321-2062744-CEBP      NNNNNNNNNNNNNNNNNNNNNNNNNNNNNNNNNNNNNNNNNNNNNNNNNNNNNNNNNNNN
                                      ************************************************************

JH171653.1||746770-749193-CEBPG2      NNNNNNNNNNNNNNNNNNNNNNNNNNNNNNNNNNNNNNNNNNNNNNNNNNNNNNNNNNNN
JH169046.1||191678-194128-CEBPG-      NNNNNNNNNNNNNNNNNNNNNNNNNNNNNNNNNNNNNNNNNNNNNNNNNNNNNNNNNNNN
JH172334.1||2060321-2062744-CEBP      NNNNNNNNNNNNNNNNNNNNNNNNNNNNNNNNNNNNNNNNNNNNNNNNNNNNNNNNNNNN
                                      ************************************************************

JH171653.1||746770-749193-CEBPG2      NNNNNNNNNNNNNNNNNNNNNNNNNNNNNNNNNNNNNNNNNNNNNNNNNNNNNNNNNNNN
JH169046.1||191678-194128-CEBPG-      NNNNNNNNNNNNNNNNNNNNNNNNNNNNNNNNNNNNNNNNNNNNNNNNNNNNNNNNNNNN
JH172334.1||2060321-2062744-CEBP      NNNNNNNNNNNNNNNNNNNNNNNNNNNNNNNNNNNNNNNNNNNNNNNNNNNNNNNNNNNN
                                      ************************************************************

JH171653.1||746770-749193-CEBPG2      NNNNNNNNNNNNNNNNNNNNNNNNNNNNNNNNNNNNNNNNNNNNNNNNNNNNNNNNNNNN
JH169046.1||191678-194128-CEBPG-      NNNNNNNNNNNNNNNNNNNNNNNNNNNNNNNNNNNNNNNNNNNNNNNNNNNNNNNNNNNN
JH172334.1||2060321-2062744-CEBP      NNNNNNNNNNNNNNNNNNNNNNNNNNNNNNNNNNNNNNNNNNNNNNNNNNNNNNNNNNNN
                                      ************************************************************

JH171653.1||746770-749193-CEBPG2      NNNNNNNNNNNNNNNNNNNNNNNNNNNNNNNNNNNNNNNNNNNNNNNNNNNNNNNNNNNN
JH169046.1||191678-194128-CEBPG-      NNNNNNNNNNNNNNNNNNNNNNNNNNNNNNNNNNNNNNNNNNNNNNNNNNNNNNNNNNNN
JH172334.1||2060321-2062744-CEBP      NNNNNNNNNNNNNNNNNNNNNNNNNNNNNNNNNNNNNNNNNNNNNNNNNNNNNNNNNNNN
                                      ************************************************************

JH171653.1||746770-749193-CEBPG2      GAAGAATTGGATATTGAAATTTAAAAGTTATGTAGGAGATTGGTCTTGGAGGCGGCAGCG
JH169046.1||191678-194128-CEBPG-      NNNNNNNNNNNNNNNNNNNNNNNNNNNNNNNNNNNNNNNNNNNNNNNNNNNNNNNNNNNN
JH172334.1||2060321-2062744-CEBP      NNNNNNNNNNNNNNNNNNNNNNNNNNNNNNNNNNNNNNNNNNNNNNNNNNNNNNNNNNNN

JH171653.1||746770-749193-CEBPG2      GCGCGCGCCGTCCCGGTGAGGAGGGAAAGCCGGTGGCCGTGGTGGCCGTGGTGGCGGAAC
JH169046.1||191678-194128-CEBPG-      NNNNNNNNNNNNNNNNNNNNNNNNNNNNNNNNNNNNNNNNNNNNNNNNNNNNNNNNNNNN
JH172334.1||2060321-2062744-CEBP      NNNNNNNNNNNNNNNNNNNNNNNNNNNNNNNNNNNNNNNNNNNNNNNNNNNNNNNNNNNN

JH171653.1||746770-749193-CEBPG2      GGGCGGCCGGTTTCGTAATCGTCCCACCTCCTAGCTGACTCCCGGGGTGGGAGACCTGGG
JH169046.1||191678-194128-CEBPG-      NNNNNNNNNNNNNNNNNNNNNNNNTACTTTCTGGCTGCCATGAACTGTTTCTCCTCTGTA
JH172334.1||2060321-2062744-CEBP      NNNNNNNNNNNNNNNNNNNNNNNTCAATTAATGGTGGCCT---GCTGTGTTAGGTACATA
                                                               *  *  * *  * *       **

JH171653.1||746770-749193-CEBPG2      TCGGGTTGGGCCGCCGCGCGGGGCCGCTCG-------GAGTGGAGGCTGCCTGGGGGCGG
JH169046.1||191678-194128-CEBPG-      -------AGGCCTGTCTGCATACTACCCTG-------CCCTGAAGTCAGCC---------
JH172334.1||2060321-2062744-CEBP      CAAATGTAAGCTCATTTGTTTGTCATTTTAAGTATTTTAATGGAGTTTGTT---------
                                               **      *                      ** **   *

JH171653.1||746770-749193-CEBPG2      GCGGGCTGGAGACGCAGTTCACTGTGGAACATCGTCTAAAACATGGAGCGACCACTAATT
JH169046.1||191678-194128-CEBPG-      --------------------------------------AATTATGGACTGAAACCTCTAT
JH172334.1||2060321-2062744-CEBP      --------------------------------------ATTTTT-----------TTTTT
                                                                            *    *           *   *

JH171653.1||746770-749193-CEBPG2      GAATAGAAAGAAACGAGTACATGTGAAGATTTTTTGGCAGTTTATTGTGGAAGCGACTGA
JH169046.1||191678-194128-CEBPG-      GAATT-----------GTACATGAGAAGTTTTTTTGGCAGTT-----TGGAAGTGACTGA
JH172334.1||2060321-2062744-CEBP      TAATAG----------GTACATGTGAAGATTTTTTGGCAGTTTATTGTGGAAGCGACTGA
                                       ***            ******* **** **************    ****** ******

JH171653.1||746770-749193-CEBPG2      TCACCCTGCTCTCATTTCTACCTGACCTGTGTTGGCAAGGGAGAGTGCCCAAATGAGCAA
JH169046.1||191678-194128-CEBPG-      TCACCTTGCCCTCATTTCTATCTGACCTGTGTTGGCAAGGGAGACTGCCTAAATGAGCAA
JH172334.1||2060321-2062744-CEBP      TCACCTTGCTCTCATTTCTACCTGACCTGTGTTGGCAAGGGAGAGTGCCCAAATGAGCAA
                                      ***** *** ********** *********************** **** **********

JH171653.1||746770-749193-CEBPG2      GATAGCGCAGCAAAACAGTAGTACTCCAGGAGTGAACGGAATAAGTGTTATTCACACTCA
JH169046.1||191678-194128-CEBPG-      GATAGCACAGCAAAAC---AGTACTCCAGGAGTGAATGGAATAAGTGTTATTCATACTCA
JH172334.1||2060321-2062744-CEBP      GATAGCGCAGCAAAACAGTAGTACTCCAGGAGTGAACGGAATAAGTGTTATTCACACTCA
                                      ****** *********   ***************** ***************** *****

JH171653.1||746770-749193-CEBPG2      GCCACATGCCAGCGGCTTGCAGCAGGTTCCTCAGCTGGTGCCTGCTGGCCCTGGGGGAGG
JH169046.1||191678-194128-CEBPG-      GGCACATGCTAGCGGCTTACAGCAGGTTCCTCAGCCTGTGCCTGCTGGCCCTAGGGAAGG
JH172334.1||2060321-2062744-CEBP      GCCACATGCCAGCGGCTTGCAGCAGGTTCCTCAGCTGGTGCCTGCTGGCCCTGGGGGAGG
                                      * ******* ******** ****************  *************** *** ***

JH171653.1||746770-749193-CEBPG2      AGGCAAAGCCGTGCCTCCAAGCAAGCAGAGCAAAAAGAGTTCACCCATGGATCGAAATAG
JH169046.1||191678-194128-CEBPG-      AGGCAAATCCATTCCTCCAAGCAAGCAGAGCAAAGAGAGTTCACCCATGGATCCAAAGAG
JH172334.1||2060321-2062744-CEBP      AGGCAAAGCCGTGCCTCCAAGCAAGCAGAGCAAAAAGAGTTCGCCCATGGATCGAAATAG
                                      ******* ** * ********************* ******* ********** *** **
```

```
JH171653.1||746770-749193-CEBPG2       TGACGAGTATCGTCAGCGCAGAGAGAGGAACAACATGGCTGTGAAAAAGAGCAGATTGAA
JH169046.1||191678-194128-CEBPG-       TAAAGAGTATCATCAGTGCAGAGAGAGGAACAACATGGCTGTGAAAAAGAGCCAATTGAA
JH172334.1||2060321-2062744-CEBP       TGACGAGTATCGTCAGCGCAGAGAGAGGAACAACATGGCTGTGAAAAAGAGCAGATTGAA
                                       * * ******* **** ***********************************  ******

JH171653.1||746770-749193-CEBPG2       AAGCAAGCAGAAAGCACAAGACACGCTGCAAAGAGTGAATCAGCTCAAAGAAGAGAATGA
JH169046.1||191678-194128-CEBPG-       AAGTAAGCAGAAAGCACAAGATATGCTGCAAAGAGTGAATCAGCTCAAAGAAGAGAATGA
JH172334.1||2060321-2062744-CEBP       AAGCAAGCAGAAAGCACAAGACACGCTGCAAAGAGTGAATCAGCTCAAAGAAGAGAATGA
                                       *** ***************** * ************************************

JH171653.1||746770-749193-CEBPG2       ACGCTTGGAAGCAAAAATTAAATTGCTGACAAAAGAATTAAGTGTACTAAAAGATTTGTT
JH169046.1||191678-194128-CEBPG-       ATGCTTGGAAGCAAAAATTCAATTGGTGACAAAGGAATTAAGTGTACTGAAAGATTTGTT
JH172334.1||2060321-2062744-CEBP       ACGCTTGGAAGCAAAAATTAAATTGCTGACAAAAGAATTAAGTGTACTAAAAGATTTGTT
                                       * ***************** ***** ******* ************** ***********

JH171653.1||746770-749193-CEBPG2       TCTTGAGCACGCACACAACCTTGCAGACAACGTGCAGCCCATTAGCACTGAAACTACGAC
JH169046.1||191678-194128-CEBPG-       TCTTGAGCATGCACACAACCTTGCAGACAATGTGAAGCCAATTAGCACTGAACATACGAC
JH172334.1||2060321-2062744-CEBP       TCTTGAGCATGCACACAACCTTGCAGACAACGTGCAGCCCATTAGCACTGAAACTACGAC
                                       ********* ******************** *** **** ************  ******

JH171653.1||746770-749193-CEBPG2       AACAAATTCTGATACTGCAGGACAGTAGACCTCAACCTTCCCAGACTTACCAGCTGTGGC
JH169046.1||191678-194128-CEBPG-       AACAAATTCTGATACTGCAGGACAGTAGACCTCAACATTCCCAGACTTCACATCTGTGGC
JH172334.1||2060321-2062744-CEBP       AACAAATTCTGATACTGCAGGACAGTAGACCTCAACCTTCCCAGACTTACCAGCTGTGGC
                                       ************************************ ***********  ** *******

JH171653.1||746770-749193-CEBPG2       TTGAACATTAAAGGTGTGACCACCTACAGCACTCAGATGTTCATGGCTGAATGCCATCTT
JH169046.1||191678-194128-CEBPG-       TTGAACATTAAAGGTGTGACTGCCTACACCACTCAGATGTCCATGGCTGAATGTCATC-T
JH172334.1||2060321-2062744-CEBP       TTGAACATTAAAGGTGTGACCACCTACAGCACTCAGATGTTCATGGCTCAATGCCATCTT
                                       ********************  ****** *********** ******* **** **** *

JH171653.1||746770-749193-CEBPG2       TTTCCATTATTCAGAGATCCATTGGAGGTTATTTTCTAGGATCAGCACTGAAGAGTTGAG
JH169046.1||191678-194128-CEBPG-       TTTCCATTATTCAAATATCCATTGGAGGTTATTTTCTAGGATTAGCACTGAAGAGTTGAT
JH172334.1||2060321-2062744-CEBP       TTTCCATTATTCAAAGATCCATTGGAGGTTATTTTCTAGGATCAGCACTGAAGAGTTGAG
                                       ************* * ************************** ****************

JH171653.1||746770-749193-CEBPG2       TAGCTGTGTCGATTAAATATATGGCTTTAAATGAGGTGGATTTTTGTGGCAATTGAAAAA
JH169046.1||191678-194128-CEBPG-       TAGCCATGTCATTTAAATATATGCCTTTAAATGAGGTGGATTTTTGTGGCAATTGAAAAA
JH172334.1||2060321-2062744-CEBP       TAGCAGTGTCGATTAAATATATGGCTTTAAATGAGGTGGATTTTTGTGGCAATTGAAAAA
                                       ****  ****  *********** ************************************

JH171653.1||746770-749193-CEBPG2       -AAAAATTTTTAAAGTATATGGTAAACATATCCTGGACAAACATATCCTGGACCTTAATG
JH169046.1||191678-194128-CEBPG-       CAAAAAATTTTAAAGTATATGGTAAA------ATAAAAAAACATGTCCTGGACTTTATTG
JH172334.1||2060321-2062744-CEBP       -AAAAATTTTTAAAGTATATGGTAAACATATCCTGGACAAACATATCCTGGACCTTAATG
                                        ***** *******************       *  * ****** ******** *** **

JH171653.1||746770-749193-CEBPG2       TTCTTAATAAGTCCTGACTTCCCCAGAAATACTTNNNNNNNNNNNNNNNNNNNNNNNNNN
JH169046.1||191678-194128-CEBPG-       TTCTTAGTAAGTCCTGACATCCCCAGAAATACTTCTTTTTTAAGTTGACAAAAGTAGTGG
JH172334.1||2060321-2062744-CEBP       TTCTTAATAAGTCCTGACTTCCCCAGAAATACTTNNNNNNNNNNNNNNNNNNNNNNNNNN
                                       ****** *********** ***************

JH171653.1||746770-749193-CEBPG2       NNNNNNNNNNNNNNNNNNNNNNNNNNNNNNNNNNNNNNNNNNNNNNNNNNNNNNNNNNNN
JH169046.1||191678-194128-CEBPG-       GAAACTGGCAGGTCATGCAGAAGGTTCTCAGTTAGAGAAAAGAAACAAATCATATACAAA
JH172334.1||2060321-2062744-CEBP       NNNNNNNNNNNNNNNNNNNNNNNNNNNNNNNNNNNNNNNNNNNNNNNNNNNNNNNNNNNN


JH171653.1||746770-749193-CEBPG2       NNNNNNNNNNNNNNNNNNNNNNNNNNNNNNNNNNNNNNNNNNNNNNNNNNNNNNNNNNNN
JH169046.1||191678-194128-CEBPG-       GGAAAGCCAATCAGAATCACAGCAGACTTCTCAATACAAACCCTCAAGGTAAGAAGAGAT
JH172334.1||2060321-2062744-CEBP       NNNNNNNNNNNNNNNNNNNNNNNNNNNNNNNNNNNNNNNNNNNNNNNNNNNNNNNNNNNN


JH171653.1||746770-749193-CEBPG2       NNNNNNNNNNNNNNNNNNNNNNNNNNNNNNNNNNNNNNNNNNNNNNNNNNNNNNNNNNNN
JH169046.1||191678-194128-CEBPG-       GCGAGTGACACATTTCAAGCCCTAAAGGAAAACAGCCTCCAATGCAGATTGATGTACCCT
JH172334.1||2060321-2062744-CEBP       NNNNNNNNNNNNNNNNNNNNNNNNNNNNNNNNNNNNNNNNNNNNNNNNNNNNNNNNNNNN


JH171653.1||746770-749193-CEBPG2       NNNNNNNNNNNNNNNNNNNNNNNNNNNNNNNNNNNNNNNNNNNNNNNNNNNNNNNNNNNN
JH169046.1||191678-194128-CEBPG-       GCAAAACTATCTTTCAAAATTGATGGAGAAATAAGATATTATCAGGACAAAGAGCACCTG
JH172334.1||2060321-2062744-CEBP       NNNNNNNNNNNNNNNNNNNNNNNNNNNNNNNNNNNNNNNNNNNNNNNNNNNNNNNNNNNN


JH171653.1||746770-749193-CEBPG2       NNNNNNNNNNNNNNNNNNNNNNNNNNNNNNNNNNNNNNNNNNNNNNNNNNNNNNNNNNNN
JH169046.1||191678-194128-CEBPG-       AGGAACGTCATGAATAACAAATCAGCACTCCAAAAAATTTTAGACAATATCTTAGGAAAA
JH172334.1||2060321-2062744-CEBP       NNNNNNNNNNNNNNNNNNNNNNNNNNNNNNNNNNNNNNNNNNNNNNNNNNNNNNNNNNNN


JH171653.1||746770-749193-CEBPG2       NNNNNNNNNNNNNNNNNNNNNNNNNNNNNNNNNNNNNNNNNNNNNNNNNNNNNNN-----
JH169046.1||191678-194128-CEBPG-       GAAAACAAGGAGAATCAGCCTGCTGCACACAGGAAGACACCTGGTTCCACACTACAAAGA
JH172334.1||2060321-2062744-CEBP       NNNNNNNNNNNNNNNNNNNNNNNNNNNNNNNNNNNNNNNNNNNNNNNNNNNNNNNNNNNN
```

```
JH171653.1||746770-749193-CEBPG2      ------------------------------------------------------------
JH169046.1||191678-194128-CEBPG-      GGTAGAGGACACCAGACCATCAGACCAAGGTACTGAGTCCCACTGCTCCTGGTCCAGAGG
JH172334.1||2060321-2062744-CEBP      NNNNNNNNNNNNNNNNNNNNNNNNNNNNNNNNNNNNNNNNNNNNNNNNNNNNNNNNNNNN

JH171653.1||746770-749193-CEBPG2      ------------------------------------------------------------
JH169046.1||191678-194128-CEBPG-      AAGGGGCTTCTGGGTCACCATCCCTCCCGCTGCTCCTGGCCCAGAGGAAGGGGCTTCTGG
JH172334.1||2060321-2062744-CEBP      NNNNNNNNNNNNNNNNNNNNNNNNNNNNNNNNNNNNNNNNNNNNNNNNNT----------

JH171653.1||746770-749193-CEBPG2      ------------------------------------------------------------
JH169046.1||191678-194128-CEBPG-      GCCACCATCCCTCCAGCTGCTCCCAGCCCACAGGAAAGGGCTTTTGGGTCACCATCCCTA
JH172334.1||2060321-2062744-CEBP      ------------------------------------------------------------

JH171653.1||746770-749193-CEBPG2      ------------------------------------------------------------
JH169046.1||191678-194128-CEBPG-      CCGCTGCTCCTGGCCCCTGTCTCACCTCACCCAGCTCAGAGGAAGTAATCCGTGCAGGCG
JH172334.1||2060321-2062744-CEBP      ------------------------------------------------------------

JH171653.1||746770-749193-CEBPG2      ------------------------------------------------------------
JH169046.1||191678-194128-CEBPG-      CTAACAGCCCCCCGCCCCAGTTGAAATGAGAAAACATGTAAATAANNNNNNNNNNNNNNN
JH172334.1||2060321-2062744-CEBP      ------------------------------------------------------------

JH171653.1||746770-749193-CEBPG2      ------------------------------------------------------------
JH169046.1||191678-194128-CEBPG-      NNNNNNNNNNNNNNNNNNNNNNNNNNNNNNNNNNNNNNNNNNNNNNNNNNNNNNNNNNNN
JH172334.1||2060321-2062744-CEBP      ------------------------------------------------------------

JH171653.1||746770-749193-CEBPG2      ------------------------------------------------------------
JH169046.1||191678-194128-CEBPG-      NNNNNNNNNNNNNNNNNNNNNNNNNNNNNNNNNNNNNNNNNNNNNNNNNNNNNNNNNNNN
JH172334.1||2060321-2062744-CEBP      ------------------------------------------------------------

JH171653.1||746770-749193-CEBPG2      ------------------------------------------------------------
JH169046.1||191678-194128-CEBPG-      NNNNNNNNNNNNNNNNNNNNNNNNNNNNNNNNNNNNNNNNNNNNNNNNNNNNNNNNNNNN
JH172334.1||2060321-2062744-CEBP      ------------------------------------------------------------

JH171653.1||746770-749193-CEBPG2      ------------------------------------------------------------
JH169046.1||191678-194128-CEBPG-      NNNNNNNNNNNNNNNNNNNNNNNNNNNNNNNNNNNNNNNNNNNNNNNNNNNNNNNNNNNN
JH172334.1||2060321-2062744-CEBP      ------------------------------------------------------------

JH171653.1||746770-749193-CEBPG2      ------------------------------------------------------------
JH169046.1||191678-194128-CEBPG-      NNNNNNNNNNNNNNNNNNNNNNNNNNNNNNNNNNNNNNNNNNNNNNNNNNNNNNNNNNNN
JH172334.1||2060321-2062744-CEBP      ------------------------------------------------------------

JH171653.1||746770-749193-CEBPG2      ------------------------------------------------------------
JH169046.1||191678-194128-CEBPG-      NNNNNNNNNNNNNNNNNNNNNNNNNNNNNNNNNNNNNNNNNNNNNNNNNNNNNNNNNNNN
JH172334.1||2060321-2062744-CEBP      ------------------------------------------------------------

JH171653.1||746770-749193-CEBPG2      ------------------------------------------------------------
JH169046.1||191678-194128-CEBPG-      NNNNNNNNNNNNNNNNNNNNNNNNNNNNNNNNNNNNNNNNNNNNNNNNNNNNNNNNNNNN
JH172334.1||2060321-2062744-CEBP      ------------------------------------------------------------

JH171653.1||746770-749193-CEBPG2      ------------------------------------------------------------
JH169046.1||191678-194128-CEBPG-      NNNNNNNNNNNNNNNNNNNNNNNNNNNNNNNNNNNNNNNNNNNNNNNNNNNNNNNNNNNN
JH172334.1||2060321-2062744-CEBP      ------------------------------------------------------------

JH171653.1||746770-749193-CEBPG2      ------------------------------------------------------------
JH169046.1||191678-194128-CEBPG-      NNNNNNNNNNNNNNNNNNNNNNNNNNNNNNNNNNNNNNNNNNNNNNNNNNNNNNNNNNNN
JH172334.1||2060321-2062744-CEBP      ------------------------------------------------------------

JH171653.1||746770-749193-CEBPG2      ------------------------------------------------------------
JH169046.1||191678-194128-CEBPG-      NNNNNNNNNNNNNNNNNNNNNNNNNNNNNNNNNNNNNNNNNNNNNNNNNNNNNNNNNNNN
JH172334.1||2060321-2062744-CEBP      ------------------------------------------------------------

JH171653.1||746770-749193-CEBPG2      ------------------------------------------------------------
JH169046.1||191678-194128-CEBPG-      NNNNNNNNNNNNNNNNNNNNNNNNNNNNNNNNNNNNNNNNNNNNNNNNNNNNNNNNNNNN
JH172334.1||2060321-2062744-CEBP      ------------------------------------------------------------
```

```
JH171653.1||746770-749193-CEBPG2      ------------------------------------------------------------
JH169046.1||191678-194128-CEBPG-      NNNNNNNNNNNNNNNNNNNNNNNNNNNNNNNNNNNNNNNNNNNNNNNNNNNNNNNNNNNN
JH172334.1||2060321-2062744-CEBP      ------------------------------------------------------------

JH171653.1||746770-749193-CEBPG2      ------------------------------------------------------------
JH169046.1||191678-194128-CEBPG-      NNNNNNNNNNNNNNNNNNNNNNNNNNNNNNNNNNNNNNNNNNNNNNNNNNNNNNNNNNNN
JH172334.1||2060321-2062744-CEBP      ------------------------------------------------------------

JH171653.1||746770-749193-CEBPG2      ------------------------------------------------------------
JH169046.1||191678-194128-CEBPG-      NNNNNNNNNNNNNNNNNNNNNNNNNNNNNNNNNNNNNNNNNNNNNNNNNNNNNNNNNNNN
JH172334.1||2060321-2062744-CEBP      ------------------------------------------------------------

JH171653.1||746770-749193-CEBPG2      ---------------------------NNNNNNNNNNNNNNNNNNNNNNNNNNNNNNNNN
JH169046.1||191678-194128-CEBPG-      NNNNNNNAGTGAAATCAATAGGACACACATGTGTAAATACTGAATAGTTTTTCTAGTGTG
JH172334.1||2060321-2062744-CEBP      ---------------------------TAATTGTAGTTAACATTTTGTTTTT--------

JH171653.1||746770-749193-CEBPG2      NNNNNNNNNNNNNNNNNNNNNNNNNNNNNNNNNNNNNNNNNNNNNNNNNNNNNNNNNNNN
JH169046.1||191678-194128-CEBPG-      AGAAACTGAAAGTGTAAATAAGGACTAAATGGTATAATAGATAATGTGAAATAGGAAGAA
JH172334.1||2060321-2062744-CEBP      ---------AAATATATATCAATTCCATACCATCTCCTAA--------------------

JH171653.1||746770-749193-CEBPG2      NNNNNNNNNNNNNNNNNNNNNNNNNNNNNNNNNNNNNNNNNNNNNNNNNNNNNNNNNNNN
JH169046.1||191678-194128-CEBPG-      AAGGGGCAAAGTGGAAGAGGAATTGGAAGGAATAGGCAAAAGCAAAGAAGGGAATGGGAT
JH172334.1||2060321-2062744-CEBP      ------------------------------------------------------------

JH171653.1||746770-749193-CEBPG2      NNNNNNNNNNNNNNNNNNNNNNNNNNNNNNNNNNNNNNNNNNNNNNNNNNNNNNNNNNNN
JH169046.1||191678-194128-CEBPG-      TAATATGAGATAATGAAAAACTGGAAACAAAATCTTTGTAAATAGGGAGGACTGGATGGG
JH172334.1||2060321-2062744-CEBP      -AACAAGAGTTTAAAAAAACTTGAAAGTGAAGGTTTT-----------------------

JH171653.1||746770-749193-CEBPG2      NNNNNNNNNNNNNNNNNNNNNNNNNNNNNNNNNNNNNNNNNNNNNNNNNNNNNNNNNNNN
JH169046.1||191678-194128-CEBPG-      TTGAAAGGAGGGCAGGGGAATGGGGAAAAATAGGAACCAAGAGAGACTATGTAAAGGTGC
JH172334.1||2060321-2062744-CEBP      ------------------------------------------------------------

JH171653.1||746770-749193-CEBPG2      NNNNNNNNNNNNNNNNNNNNNNNNNNNNNNNNNNNNNNNNNNNNNNNNNNNNNNNNNNNN
JH169046.1||191678-194128-CEBPG-      AAATTTCCAGTGATGAATGTCATTATATTGTATATACAATGTTCACCAATAAAATAAAAT
JH172334.1||2060321-2062744-CEBP      --------------------------------------------------------AACC

JH171653.1||746770-749193-CEBPG2      NNNNNNNNNNNNNNNNNNNNNNNNNNNNNNNNNNNNNNNNNNNNNNNNNNNNNNNNNNNN
JH169046.1||191678-194128-CEBPG-      TTTAAAAAATAATTTNNNNNNNNNNNNNNNNNNNNNNNNNNNNNNNNNNNNNNNNNNNNN
JH172334.1||2060321-2062744-CEBP      TTTAA-------------------------------------------------------

JH171653.1||746770-749193-CEBPG2      NNNNNNNNNNNNNNNNNNNNNNNNNNNNNNNNNNNNNNNNNNNNNNNNNNNNNNNNNNNN
JH169046.1||191678-194128-CEBPG-      NNNNNNNNNNNNNNNNNNNNNNNNNNNNNNNNNNNNNNNNNNNNNNNNNNNATCTCTTTA
JH172334.1||2060321-2062744-CEBP      --------------------------------------------------------TTTA

JH171653.1||746770-749193-CEBPG2      NNNNNNNNNNNNNNNNNNNNNNNNNNNNNNNNNNNNNNNNNNNNNNNNNNNNNNNNNNNN
JH169046.1||191678-194128-CEBPG-      TGTTTTTTGGGTTTTTTTTTTTTTTTTTTTTTTTTTTTTTTGGTGACAATTCTTTTAGTT
JH172334.1||2060321-2062744-CEBP      TTTTGCTTAAATACATCTGTTTATGTTTTGGGATTTTTTTTGGTGACAGTTCTTTTAGTT

JH171653.1||746770-749193-CEBPG2      NNNNNNNNNNNNNNNNNNNNNNNNNNNNNNNNNNNNNNNNNNNNNNNNNNNNNNNNNNNN
JH169046.1||191678-194128-CEBPG-      AGTGCACTGTTCTTCCAGACTTTATGTACCACTGCTTCTGTTCATTCATTGTGCGCTTTT
JH172334.1||2060321-2062744-CEBP      AGTGGGCTG---TTCCCGACTTTGTGTACCACTGCTTCTTTTGATTCATTGTTTGCTTTT

JH171653.1||746770-749193-CEBPG2      NNNNNNNNNNNNNNNNNNNNNNNNNNNNNNNNNNNNNNNNNNNNNNNNNNNNNNNNNNNN
JH169046.1||191678-194128-CEBPG-      ATGTTCCATAAATTATTTTGGAAATAAAATGCTGGTAAAACTCAGGATATTGATATTTTT
JH172334.1||2060321-2062744-CEBP      ATGTCCCATAAATTATTTTGGAAAGAAAATGCTGGTAAAACTCAGGATATTGACATTTTT

JH171653.1||746770-749193-CEBPG2      NNNNNNNNNNNNNNNNNNNNNNNNNNNNNNNNNNNNNNNNNNNNNNNNNNNNNNNNNNNN
JH169046.1||191678-194128-CEBPG-      GTGGAGAC-AAAAAAATGGCAATCACTAATGTAGGGCACCTCTAGAATCTGCGTTATATC
JH172334.1||2060321-2062744-CEBP      GTGGAGACAAAAAAAAATGGCAATCACTAATGTAGGGCACCTCTAGAGTCT--GTTATATC

JH171653.1||746770-749193-CEBPG2      NNNNNNNNNNNNNNNNNNNNNNNNNNNNNNNNNNNNNNNNNNNNNNNNNNNNNNNNNNNN
JH169046.1||191678-194128-CEBPG-      AGTGTTGGTAGTTTAGATTGTTAACTTTGTCTTACAGCATGATTCTAACTCAAGTCTGTT
JH172334.1||2060321-2062744-CEBP      AGTGTTGGCAGTTTAGATTGTTAACTTTGTCTTAGAGCATGATGCTAGTTCAAGTCTATT
```

```
JH171653.1||746770-749193-CEBPG2      NNNNNNNNNNNNNNNNNNNNNNNNNNNNNNNNNNNNNNNNNNNNNNNNNNNNNNNNNNNN
JH169046.1||191678-194128-CEBPG-      GACTTCTCTGTATTGTTTTGTGCCTGCAATGGTGATTAGACAGTCAGACTTGATTAAGGT
JH172334.1||2060321-2062744-CEBP      GGCTTCTGTGTATTGTCTTGTGCCTGGGATGGTGATTAGACAGTCAGACGTGATTAAGGT

JH171653.1||746770-749193-CEBPG2      NNNNNNNNNNNNNNNNNNNNNNNNNNNNNNNNNNNNNNNNNNNNNNNNNNNNNNNNNNNN
JH169046.1||191678-194128-CEBPG-      TTCTAATTTGAATGTGGTCATTTCTGACCACACTGCTGTTTCCTACTAGGACTTTGTGAT
JH172334.1||2060321-2062744-CEBP      TTCTTATTTGAATGTGGCCATTTCTGGCCACACTGCTGTTTCCTACTAGGAC-TTGGGAT

JH171653.1||746770-749193-CEBPG2      NNNNNNNNNNNNNNNNNNNNNNNNNNNNNNNNNNNNNNNNNNNNNNNNNNNNNNNNNNNN
JH169046.1||191678-194128-CEBPG-      AATGTAACCAGACTGTAACTCAAGCTGTACCCTGGGTTTGGTGGAAGTGTGCTGACTGTG
JH172334.1||2060321-2062744-CEBP      AATGTAACCAGACTGTAACTCAAGCTGCACCCTGGGTTTGGTGGAAGTGTGCTGACTGTG

JH171653.1||746770-749193-CEBPG2      NNNNNNNNNNNNNNNNNNNNNNNNNNNNNNNNNNNNNNNNNNNNNNNNNNNNNNNNNNNN
JH169046.1||191678-194128-CEBPG-      CTATTCTCCTGGGCAGAAGGCAACATAGTGCTCTGTAACTCAGCAAAGATTTTCAGGGTT
JH172334.1||2060321-2062744-CEBP      CTCTTCTCCTGGGCAGAAGATGACATAGTGCCCTGCAACTCAGCAGAGATTTTTAGGGCT

JH171653.1||746770-749193-CEBPG2      NNNNNNNNNNNNNNNNNNNNNNNNNNNNNNNNNNNNNNNNNNNNNNNNNNNNNNNNNNNN
JH169046.1||191678-194128-CEBPG-      ACAAAGTCCTTGTAAATGTGGGATTGGAGCCAGTTGTTGTACCATGAG-ATACTTCCCTG
JH172334.1||2060321-2062744-CEBP      ACAAGGTCCTTGTAAATGTGGGATTGGAGCCAGGTGCTGTTCCATGAACCTACTTCCCTG

JH171653.1||746770-749193-CEBPG2      NNNNNNNNNNNNNNNNNNNNNNNNNNNNNNNNNNNNNNNNNNNNNNNNNNNNNNNNNNNN
JH169046.1||191678-194128-CEBPG-      GAGGGCTATTAATTCCCAATTCAGCCAAGAAATGACCGGGATTTTTTCTTCTTGAGTTAG
JH172334.1||2060321-2062744-CEBP      GAGGGCTATTAATTCCCAATACAATCAAGAAATGGTAGATTTTTTTCCTTTTTAAGTTAG

JH171653.1||746770-749193-CEBPG2      NNNNNNNNNNNNNNNNNNNNNNNNNNNNNNNNNNNNNNNNNNNNNNNNNNNNNNNNNNNN
JH169046.1||191678-194128-CEBPG-      GGTCCAACTGCAGTTTACTTTGGTTGGAATATTGATCTACTCATAGGGGTTGATACATGT
JH172334.1||2060321-2062744-CEBP      GGTCCAACAGCAGTTTACTTTGGTTGGAATATCGATCTACTCATAGGGGTTGATAGATAT

JH171653.1||746770-749193-CEBPG2      NNNNNNNNNNNNNNNNNNNNNNNNNNNNNNNNNNNNNNNNNNNNNNNNNNNNNNNNNNNN
JH169046.1||191678-194128-CEBPG-      TCAAATAACTACCACTTACCAACAGAGTCCTACTAAATCTTAACAGCTGATTGATGTTGT
JH172334.1||2060321-2062744-CEBP      TCAAATAACTAGGACTTACCAACACAGTCCTACTAAATC-TAACAGCTGATTGATGTTGT

JH171653.1||746770-749193-CEBPG2      NNNNNNNNNNNNNNNNNNNNNNNNNNNNNNNNNNNNNNNNNNNNNNNNNNNNNNNNNNNN
JH169046.1||191678-194128-CEBPG-      ATAATTAATTGGAGATGTAGTTAGGCCACTTGAATCCTTAAGCACCTATAATAGGTGTTA
JH172334.1||2060321-2062744-CEBP      ATAATTAATTGGAGATGTAGTTAGCCCACTTGAATCTTTAAACACCTATAATAGGTGTTA

JH171653.1||746770-749193-CEBPG2      NNNNNNNNNNNNNNNNNNNNNNNNNNNNNNNNNNNNNNNNNNNNNNNNNNNNNNNNNNNN
JH169046.1||191678-194128-CEBPG-      CATTTTAATCCCTTATTGAAAAAAAGTCAGAAATGGATGTCCAGATGGAATTTGTGGTTA
JH172334.1||2060321-2062744-CEBP      TATTTTAATCCCTTATTTAAAAAAA-TCAGAAATGGATGTCTACATGAAATTTGTGGTTA

JH171653.1||746770-749193-CEBPG2      NNNNNNNNNNNNNNNNNNNNNNNNNNNNNNNNNNNNNNNNNNNNNNNNNNNNNNNNNNNN
JH169046.1||191678-194128-CEBPG-      CCTTTCAATGATATGGAAAAAAAGCT----------------------------------
JH172334.1||2060321-2062744-CEBP      C----CAGTGATATG--AAAAAAGCTGTAGGNNNNNNNNNNNNNNNNNNNNNNNNNNNNN

JH171653.1||746770-749193-CEBPG2      NNNNNNNNNNNNGTAGGGAA---ATCAAGAGTGTGTACTCAGT-----------------
JH169046.1||191678-194128-CEBPG-      ------------GAAGGGAAATCATCAAGAGTGTGTACTCAGTGTTTCAAGTCAGAACAT
JH172334.1||2060321-2062744-CEBP      NNNNNNNNNNNNGTAGGGAA---ATCAAGAGTGTGTACTCAGT-----------------
                                                  * ******   ********************

JH171653.1||746770-749193-CEBPG2      ----------GTTTGAGAAAAATTGATTCAAGCTTTCCTATTACCTATGCAAATCAGAAT
JH169046.1||191678-194128-CEBPG-      AAAATTGGGAGTTTGAGAAAAATTGACTCAAGATTTCCTTTTAGCTATGCAAATCAGAAT
JH172334.1||2060321-2062744-CEBP      ----------GTTTGAGAAAAATTGATTCAAGCTTTCCTATTACCTATGCAAATCAGAAT
                                                ****************  ***** ****** *** ****************

JH171653.1||746770-749193-CEBPG2      TGAACACAAGCAGTCATGCATAGAACAAAAATGTTAACCATTGTCTTCACTATTTTATTC
JH169046.1||191678-194128-CEBPG-      TGAACACAAGCAGTCATGCTTAGAAGAAAAATATTAACCATTATCTTGGCTATTTTAATC
JH172334.1||2060321-2062744-CEBP      TGAACACGAGCAGTCATGCATAGAACAAAAACGTTAACCATTATCTTCACTATTTTATTC
                                      ******* *********** ***** *****  ********* ****  ******** **

JH171653.1||746770-749193-CEBPG2      AAGTGGTTAAGAATGGAAGTGTTTCGTTTGGTAGATTTAGCTACCAAAAGTGAGAGTACA
JH169046.1||191678-194128-CEBPG-      AAGTGGTTAAGAATGGATATTTTTAGTTTGGTAGATTTAGCTACCAAAATTGAGAGTACA
JH172334.1||2060321-2062744-CEBP      AAGTGGTTAAGAATGGAAGTTTTTAGTTTGGTAGATTTAGCTACCAAAAGTGAGAGTACA
                                      *****************  * *** ************************ **********

JH171653.1||746770-749193-CEBPG2      GAAATTGAGAGAATTCAAGTATCGAGAGTGAGTAAGTTTAGAGTTTTGGGAATTGTAAAG
JH169046.1||191678-194128-CEBPG-      GAAATTGAGAGAAATCAAGGGTCTGGAGTGAATAAGTTTAGAGCTTTGAGAATTGTAAAG
JH172334.1||2060321-2062744-CEBP      GAAATTGAGAGAATTCAAGTATCGAGAGTGAGTAAGTTTAGAGTTTTGGGAATTGTAAAG
                                      ************* *****  **  ****** *********** **** ***********
```

```
JH171653.1||746770-749193-CEBPG2      TGACTGTTGTAACTTTGGTTGTGAGTTTGTAACTTAGGAACCATTCGGATTTGATCTGTT
JH169046.1||191678-194128-CEBPG-      TGACTGTTGTAATTTTGGTTGTGAGCTTGTAACTTAGGAACCATTCGGATTTGATCTGCT
JH172334.1||2060321-2062744-CEBP      TGACTGTTGTAACTTTGGTTGTGAGTTTGTAACTTAGGAACCATTCGGATTTGATCTGTT
                                      ************ ************ ******************************** *

JH171653.1||746770-749193-CEBPG2      TTGCATCACTTTGGGTGGTTAAGGATTAGGCT-TAGTAAAAGTTGCTAGCTGCATCTCGG
JH169046.1||191678-194128-CEBPG-      TTGCATCAGGTCTGGTGGTTAGGGATTAGGCTGTAGTATAAGTTGCTAGCTGCATCTCGG
JH172334.1||2060321-2062744-CEBP      TTGCATCACTTTGGGTGGTTAAGGATTAGGCT-TAGTAAAAGTTGCTAGCTGCATCTCGG
                                      ********  *  ******** ********** ***** *********************

JH171653.1||746770-749193-CEBPG2      AAGG----TGCCTTTAGACTGGTTGAGGCTGCAGATGGTGACAGCAGAGCAGCTTTTCCT
JH169046.1||191678-194128-CEBPG-      ATGGATGTTGCGTTTTGACTGGCTGAGGCTGCAGATGGTGAGAGCAGAGCAGCTCTTCCC
JH172334.1||2060321-2062744-CEBP      AAGG----TGCCTTTAGACTGGTTGAGGCTGCAGATGGTGACAGCAGAGCAGCTTTTCCT
                                      * **    *** *** ****** ****************** ************ ****

JH171653.1||746770-749193-CEBPG2      GTGTCTGGCCCCTTTCAGTTGTTGGGATGCCAGTTCATATGCTTTGGACA---TTACTAA
JH169046.1||191678-194128-CEBPG-      ATGTCTGGCCCCTTTCAGTTGTTGGGATGCCAGTTCAGATGCTTTGGACATTCTTACTAA
JH172334.1||2060321-2062744-CEBP      GTGTCTGGCCCCTTTCAGTTGTTGGGATGCCAGTTCATATGCTTTGGACA---TTACTAA
                                       ************************************ ************   *******

JH171653.1||746770-749193-CEBPG2      GAATTAGTAAGACTATAAAATATGATTCAATTTTTAAAATTCAGGTCATGAGTGTAAAAT
JH169046.1||191678-194128-CEBPG-      GAATTAGTAGGACTATAAAATGTGACTCGATTTTTGAAATTCAGGTCATGAGTGCAAAAT
JH172334.1||2060321-2062744-CEBP      GAATTAGTAAGACTATAAAATATGACTCAATTTTTAAAATTCAGGTCATGAGTGTAAAAT
                                      ********* *********** *** ** ****** ****************** *****

JH171653.1||746770-749193-CEBPG2      TCTCAAATTTATGTAAAAAGTAGAAGTATGTACAATTTGATATTAAAAGAAAAGAAACCG
JH169046.1||191678-194128-CEBPG-      ACACAAATTTATGTAAAAAGTAGAAGTATGTACAATTTGATATTAAAAGAAAGGAAAATG
JH172334.1||2060321-2062744-CEBP      TCTCAAATTTATGTAAAAAGTAGAAGTATGTACAATTTGATATTAAAAGAAAGGAAACCG
                                       * ************************************************* ****  *

JH171653.1||746770-749193-CEBPG2      TAGCAGCTTTTGATGTTTCTTAATCCCCGTTAGAGGGCTGAAACTTTGTACCTGAACAAC
JH169046.1||191678-194128-CEBPG-      TAGCAGCTTTTGACGTTTCTTAATCCCCATTAGAGGGCTGAAATTTTGTACCTGAACAAC
JH172334.1||2060321-2062744-CEBP      TAGCAGCTTTTGATGTTTCTTAATCCCCATTAGAGGGCTGAAACTTTGTACCTGAAGAAC
                                      ************* ************** ************** ************ ***

JH171653.1||746770-749193-CEBPG2      TCATTTAGCACTCAGTGCTTTCTGACACCTTAGAAATAGGAAAAGTAATTTTTGTTTGAC
JH169046.1||191678-194128-CEBPG-      CCATTTAGCACTCAGTGCTTTTTGATGCCTTAGGAATAGGAAAAGTAATTTTTGTTTGAC
JH172334.1||2060321-2062744-CEBP      CCATTTAGCACTCAGTGCTTTCTGATGCCTTAGAAATAGGAAAAGTAATTTTTGTTTGAC
                                       ******************** ***  ****** **************************

JH171653.1||746770-749193-CEBPG2      ACAGGGTAGGAAAGAGAAAGTCCATTCTACAGCTAATTAGATCTGCCTCTCAGGAAAAAG
JH169046.1||191678-194128-CEBPG-      AAAAGGTAGGAAAGAGAAAGTCCCTTCTACAGCTCATTAGATCTGCCTCTCAGAAAAAAG
JH172334.1||2060321-2062744-CEBP      ACAAGGTAGGAAAGAGAAAGTCCATTCTATGGCTAATTAGATCTGCCTCTCAGGAAAAAG
                                      * * ******************* *****  *** ****************** ******

JH171653.1||746770-749193-CEBPG2      TACTAACTTGTTCTTTTTGTTCCTGGCTTTCAGTTTGTGAGATTACTCTATTTTTTTAAT
JH169046.1||191678-194128-CEBPG-      TACTAACTTGTTCTTTTTGTTCTTGGCTTTCAGTTTGTGAGATTACTCTA--TTTTTAAT
JH172334.1||2060321-2062744-CEBP      TACTAACTTGTTCTTTTTGTTCCTGGCTTTCAGTTTGTGAGATTACTCTATTTTTTTAAT
                                      ********************** ***************************  ********

JH171653.1||746770-749193-CEBPG2      ATAATTTTATTTCTTTCAATAAATGTTGAAATAAAAACTTTTTGGAACAAATAAATAAAT
JH169046.1||191678-194128-CEBPG-      GTAATTTTATTTTTTTCAATAAATCCTAAAAT---AATTTTTTGGAACAAAAAAATAATG
JH172334.1||2060321-2062744-CEBP      ATAATTTTATTTCTTTCAATAAATCTTAAAATAAAAACTTTTTGGAACAATGA-------
                                       *********** ***********  * ****   ** ************  *

JH171653.1||746770-749193-CEBPG2      AAATAAATAAATAAATAAAAATAA---AAGTTATGTAGGAGTAAAAAAGACCTAGAAAAG
JH169046.1||191678-194128-CEBPG-      AGTGATAGTGATATCTCATTGTGG---TTTTAACCTGTATTTTCAGAAGGCCCACTGATA
JH172334.1||2060321-2062744-CEBP      --------CTGTATTTCATCGTGGGTTTCTTTACATTAGATAACAAAAAGCACATGTAAA
                                                 **  * *   *        * *  *        * **  *  *   *

JH171653.1||746770-749193-CEBPG2      TAAATAGGAAAAAAA---------------------------------------------
JH169046.1||191678-194128-CEBPG-      CTCCANNNNNNNNNNNNNNNNNNNNNNNNNNNNNNNNNNNNNNNNNNNNNNNNNNNNNNN
JH172334.1||2060321-2062744-CEBP      CAGCTCTGAAGAGCATGCAGGCTTGGCTGTCTCCTGTTGCCTGGGGCGGGGGTGAGGGGT


JH171653.1||746770-749193-CEBPG2      -AAAACATTGTTGGAAGATNNNNNNNNNNNNNNNNNNNNNNNNNNNNNNNNNNNNNNNNN
JH169046.1||191678-194128-CEBPG-      NNNNNNNNNNNNNNNNNNNNNNNNNNNNNNNNNNNNNNNNNNNNNNNNNNNNNNNNNNNN
JH172334.1||2060321-2062744-CEBP      GGAGGTGCTTTTGNNNNNNNNNNNNNNNNNNNNNNNNNNNNNNNNNNNNNNNNNNNNNNN
                                                         *****************************************

JH171653.1||746770-749193-CEBPG2      NNNNNNNNNNNNNNNNNNNNNNNNNNNNNNNNNNNNNNNNNNNNNNNNNNNNNNNNNNNN
JH169046.1||191678-194128-CEBPG-      NNNNNNNNNNNNNNNNNNNNNNNNNNNNNNNNNNNNNNNNNNNNNNNNNNNNNNNNNNNN
JH172334.1||2060321-2062744-CEBP      NNNNNNNNNNNNNNNNNNNNNNNNNNNNNNNNNNNNNNNNNNNNNNNNNNNNNNNNNNNN
                                      ************************************************************

JH171653.1||746770-749193-CEBPG2      NNNNNNNNNNNNNNNNNNNNNNNNNNNNNNNNNNNNNNNNNNNNNNNNNNNNNNNNNNNN
JH169046.1||191678-194128-CEBPG-      NNNNNNNNNNNNNNNNNNNNNNNNNNNNNNNNNNNNNNNNNNNNNNNNNNNNNNNNNNNN
JH172334.1||2060321-2062744-CEBP      NNNNNNNNNNNNNNNNNNNNNNNNNNNNNNNNNNNNNNNNNNNNNNNNNNNNNNNNNNNN
                                      ************************************************************
```

```
JH171653.1||746770-749193-CEBPG2      NNNNNNNNNNNNNNNNNNNNNNNNNNNNNNNNNNNNNNNNNNNNNNNNNNNNNNNNNNNN
JH169046.1||191678-194128-CEBPG-      NNNNNNNNNNNNNNNNNNNNNNNNNNNNNNNNNNNNNNNNNNNNNNNNNNNNNNNNNNNN
JH172334.1||2060321-2062744-CEBP      NNNNNNNNNNNNNNNNNNNNNNNNNNNNNNNNNNNNNNNNNNNNNNNNNNNNNNNNNNNN
                                      ************************************************************

JH171653.1||746770-749193-CEBPG2      NNNNNNNNNNNNNNNNNNNNNNNNNNNNNNNNNNNNNNNNNNNNNNNNNNNNNNNNNNNN
JH169046.1||191678-194128-CEBPG-      NNNNNNNNNNNNNNNNNNNNNNNNNNNNNNNNNNNNNNNNNNNNNNNNNNNNNNNNNNNN
JH172334.1||2060321-2062744-CEBP      NNNNNNNNNNNNNNNNNNNNNNNNNNNNNNNNNNNNNNNNNNNNNNNNNNNNNNNNNNNN
                                      ************************************************************

JH171653.1||746770-749193-CEBPG2      NNNNNNNNNNNNNNNNNNNNNNNNNNNNNNNNNNNNNNNNNNNNNNNNNNNNNNNNNNNN
JH169046.1||191678-194128-CEBPG-      NNNNNNNNNNNNNNNNNNNNNNNNNNNNNNNNNNNNNNNNNNNNNNNNNNNNNNNNNNNN
JH172334.1||2060321-2062744-CEBP      NNNNNNNNNNNNNNNNNNNNNNNNNNNNNNNNNNNNNNNNNNNNNNNNNNNNNNNNNNNN
                                      ************************************************************

JH171653.1||746770-749193-CEBPG2      NNNNNNNNNNNNNNNNNNNNNNNNNNNNNNNNNNNNNNNNNNNNNNNNNNNNNNNNNNNN
JH169046.1||191678-194128-CEBPG-      NNNNNNNNNNNNNNNNNNNNNNNNNNNNNNNNNNNNNNNNNNNNNNNNNNNNNNNNNNNN
JH172334.1||2060321-2062744-CEBP      NNNNNNNNNNNNNNNNNNNNNNNNNNNNNNNNNNNNNNNNNNNNNNNNNNNNNNNNNNNN
                                      ************************************************************

JH171653.1||746770-749193-CEBPG2      NNNNNNNNNNNNNNNNNNNNNNNNNNNNNNNNNNNNNNNNNNNNNNNNNNNNNNNNNNNN
JH169046.1||191678-194128-CEBPG-      NNNNNNNNNNNNNNNNNNNNNNNNNNNNNNNNNNNNNNNNNNNNNNNNNNNNNNNNNNNN
JH172334.1||2060321-2062744-CEBP      NNNNNNNNNNNNNNNNNNNNNNNNNNNNNNNNNNNNNNNNNNNNNNNNNNNNNNNNNNNN
                                      ************************************************************

JH171653.1||746770-749193-CEBPG2      NNNNNNNNNNNNNNNNNNNNNNNNNNNNNNNNNNNNNNNNNNNNNNNNNNNNNNNNNNNN
JH169046.1||191678-194128-CEBPG-      NNNNNNNNNNNNNNNNNNNNNNNNNNNNNNNNNNNNNNNNNNNNNNNNNNNNNNNNNNNN
JH172334.1||2060321-2062744-CEBP      NNNNNNNNNNNNNNNNNNNNNNNNNNNNNNNNNNNNNNNNNNNNNNNNNNNNNNNNNNNN
                                      ************************************************************

JH171653.1||746770-749193-CEBPG2      NNNNNNNNNNNNNNNNNNNNNNNNNNNNNNNNNNNNNNNNNNNNNNNNNNNNNNNNNNNN
JH169046.1||191678-194128-CEBPG-      NNNNNNNNNNNNNNNNNNNNNNNNNNNNNNNNNNNNNNNNNNNNNNN-------------
JH172334.1||2060321-2062744-CEBP      NNNNNNNNNNNNNNNNNNNNNNNNNNNNNNNNNNNNNNNNNNNNNNNNNNNNNNNNNNNN
                                      ***********************************************

JH171653.1||746770-749193-CEBPG2      NNNNNNNNNNNNNNNNNNNNNNNNNNNNNNNNNNNNNNNNNNNNNNNNNNNNNNNNNNNN
JH169046.1||191678-194128-CEBPG-      ------------------------------------------------------------
JH172334.1||2060321-2062744-CEBP      NNNNNNNNNNNNNNNNNNNNNNNNNNNNNNNNNNNNNNNNNNNNNNNNNNNNNNNNNNNN


JH171653.1||746770-749193-CEBPG2      NNNNNNN
JH169046.1||191678-194128-CEBPG-      -------
JH172334.1||2060321-2062744-CEBP      NNNNNNN
```

Supplementary Figure 5. Alignment of RNA-seq reads to copies of CEBPG gene in NMR shows expression in two copies and none in the third copy.

TINF2

denotes evidence of transcription

```
CLUSTAL W (1.81) multiple sequence alignment

JH168072:139551-140968-TINF2-1      NNNNNNNNNNNNNNNNNNNNNNNNNNNNNNNNNNNNNNNNNNNNNNNNNNNNNNNNNNNN
JH168072:187433-188825-TINF2-2      NNNNNNNNNNNNNNNNNNNNNNNNNNNNNNNNNNNNNNNNNNNNNNNNNNNNNNNNNNNN
                                    ************************************************************

JH168072:139551-140968-TINF2-1      NNNNNNNNNNNNNNNNNNNNNNNNNNNNNNNNNNNNNNNNNNNNNNNNNNNNNNNNNNNN
JH168072:187433-188825-TINF2-2      NNNNNNNNNNNNNNNNNNNNNNNNNNNNNNNNNNNNNNNNNNNNNNNNNNNNNNNNNNNN
                                    ************************************************************

JH168072:139551-140968-TINF2-1      NNNNNNNNNNNNNNNNNNNNNNNNNNNNNNNNNNNNNNNNNNNNNNNNNNNNNNNNNNNN
JH168072:187433-188825-TINF2-2      NNNNNNNNNNNNNNNNNNNNNNNNNNNNNNNNNNNNNNNNNNNNNNNNNNNNNNNNNNNN
                                    ************************************************************

JH168072:139551-140968-TINF2-1      NNNNNNNNNNNNNNNNNNNNNNNNNNNNNNNNNNNNNNNNNNNNNNNNNNNNNNNNNNNN
JH168072:187433-188825-TINF2-2      NNNNNNNNNNNNNNNNNNNNNNNNNNNNNNNNNNNNNNNNNNNNNNNNNNNNNNNNNNNN
                                    ************************************************************

JH168072:139551-140968-TINF2-1      NNNNNNNNNNNNNNNNNNNNNNNNNNNNNNNNNNNNNNNNNNNNNNNNNNNNNNNNNNNN
JH168072:187433-188825-TINF2-2      NNNNNNNNNNNNNNNNNNNNNNNNNNNNNNNNNNNNNNNNNNNNNNNNNNNNNNNNNNNN
                                    ************************************************************

JH168072:139551-140968-TINF2-1      NNNNNNNNNNNNNNNNNNNNNNNNNNNNNNNNNNNNNNNNNNNNNNNNNNNNNNNNNNNN
JH168072:187433-188825-TINF2-2      NNNNNNNNNNNNNNNNNNNNNNNNNNNNNNNNNNNNNNNNNNNNNNNNNNNNNNNNNNNN
                                    ************************************************************

JH168072:139551-140968-TINF2-1      NNNNNNNNNNNNNNNNNNNNNNNNNNNNNNNNNNNNNNNNNNNNNNNNNNNNNNNNNNNN
JH168072:187433-188825-TINF2-2      NNNNNNNNNNNNNNNNNNNNNNNNNNNNNNNNNNNNNNNNNNNNNNNNNNNNNNNNNNNN
                                    ************************************************************

JH168072:139551-140968-TINF2-1      NNNNNNNNNNNNNNNNNNNNNNNNNNNNNNNNNNNNNNNNNNNNNNNNNNNNNNNNNNNN
JH168072:187433-188825-TINF2-2      NNNNNNNNNNNNNNNNNNNNNNNNNNNNNNNNNNNNNNNNNNNNNNNNNNNNNNNNNNNN
                                    ************************************************************

JH168072:139551-140968-TINF2-1      NNNNNNNNNNNNNNNNNNNNNNNNNNNNNNNNNNNNNNNNNNNNNNNNNNNNNNNNNNNN
JH168072:187433-188825-TINF2-2      NNNNNNNNNNNNNNNNNNNNNNNNNNNNNNNNNNNNNNNNNNNNNNNNNNNNNNNNNNNN
                                    ************************************************************

JH168072:139551-140968-TINF2-1      NNNNNNNNNNNNNNNNNNNNNNNNNNNNNNNNNNNNNNNNNNNNNNNNNNNNNNNNNNNN
JH168072:187433-188825-TINF2-2      NNNNNNNNNNNNNNNNNNNNNNNNNNNNNNNNNNNNNNNNNNNNNNNNNNNNNNNNNNNN
                                    ************************************************************

JH168072:139551-140968-TINF2-1      NNNNNNNNNNNNNNNNNNNNNNNNNNNNNNNNNNNNNNNNNNNNNNNNNNNNNNNNNNNN
JH168072:187433-188825-TINF2-2      NNNNNNNNNNNNNNNNNNNNNNNNNNNNNNNNNNNNNNNNNNNNNNNNNNNNNNNNNNNN
                                    ************************************************************

JH168072:139551-140968-TINF2-1      NNNNNNNNNNNNNNNNNNNNNNNNNNNNNNNNNNNNNNNNNNNNNNNNNNNNNNNNNNNN
JH168072:187433-188825-TINF2-2      NNNNNNNNNNNNNNNNNNNNNNNNNNNNNNNNNNNNNNNNNNNNNNNNNNNNNNNNNNNN
                                    ************************************************************

JH168072:139551-140968-TINF2-1      NNNNNNNNNNNNNNNNNNNNNNNNNNNNNNNNNNNNNNNNNNNNNNNNNNNNNNNNNNNN
JH168072:187433-188825-TINF2-2      NNNNNNNNNNNNNNNNNNNNNNNNNNNNNNNNNNNNNNNNNNNNNNNNNNNNNNNNNNNN
                                    ************************************************************

JH168072:139551-140968-TINF2-1      NNNNNNNNNNNNNNNNNNNNNNNNNNNNNNNNNNNNNNNNNNNNNNNNNNNNNNNNNNNN
JH168072:187433-188825-TINF2-2      NNNNNNNNNNNNNNNNNNNNNNNNNNNNNNNNNNNNNNNNNNNNNNNNNNNNNNNNNNNN
                                    ************************************************************

JH168072:139551-140968-TINF2-1      NNNNNNNNNNNNNNNNNNNNNNNNNNNNNNNNNNNNNNNNNNNNNNNNNNNNNNNNNNNN
JH168072:187433-188825-TINF2-2      NNNNNNNNNNNNNNNNNNNNNNNNNNNNNNNNNNNNNNNNNNNNNNNNNNNNNNNNNNNN
                                    ************************************************************

JH168072:139551-140968-TINF2-1      NNNNNNNNNNNNNNNNNNNNNNNNNNNNNNNNNNNNNNNNNNNNNNNNNNNNNNNNNNNN
JH168072:187433-188825-TINF2-2      NNNNNNNNNNNNNNNNNNNNNNNNNNNNNNNNNNNNNNNNNNNNNNNNNNNNNNNNNNNN
                                    ************************************************************

JH168072:139551-140968-TINF2-1      NNNNNNNNNNNNNNNNNNNNNNNNNNNNNNNNNNNNNNNNNNNNNNNNNNNNNNNNNNNN
JH168072:187433-188825-TINF2-2      NNNNNNNNNNNNNNNNNNNNNNNNNNNNNNNNNNNNNNNNNNNNNNNNNNNNNNNNNNNN
                                    ************************************************************

JH168072:139551-140968-TINF2-1      NNNNNNNNNNNNNNNNNNNNNNNNNNNNNNNNNNNNNNNNNNNNNNNNNNNNNNNNNNNN
JH168072:187433-188825-TINF2-2      NNNNNNNNNNNNNNNNNNNNNNNNNNNNNNNNNNNNNNNNNNNNNNNNNNNNNNNNNNNN
                                    ************************************************************
```

```
JH168072:139551-140968-TINF2-1      NNNNNNNNNNNNNNNNNNNNNNNNNNNNNNNNNNNNNNNNNNNNNNNNNNNNNNNNNNNN
JH168072:187433-188825-TINF2-2      NNNNNNNNNNNNNNNNNNNNNNNNNNNNNNNNNNNNNNNNNNNNNNNNNNNNNNNNNNNN
                                    ************************************************************

JH168072:139551-140968-TINF2-1      NNNNNNNNNNNNNNNNNNNNNNNNNNNNNNNNNNNNNNNNNNNNNNNNNNNNNNNNNNNN
JH168072:187433-188825-TINF2-2      NNNNNNNNNNNNNNNNNNNNNNNNNNNNNNNNNNNNNNNNNNNNNNNNNNNNNNNNNNNN
                                    ************************************************************

JH168072:139551-140968-TINF2-1      ATGGCCACGCCCCAGGGGGCCGGTTCCGCCACTTGGCGCTTGGCGGCCGCAGCCTGCTGG
JH168072:187433-188825-TINF2-2      ATGGCCACGCCCCAGGGGGCCGGTCCCGCCACTTGGCGCTTGGCGGCCGCAGCCTGCTGG
                                    ************************ ***********************************

JH168072:139551-140968-TINF2-1      CGAGTGGTGCGCGGACGCTGCGTGGAGCATTTCCCGCGAGTCCTGCAGTTTCTGCGCTCC
JH168072:187433-188825-TINF2-2      CGAGTGGTGCGCGGACGCTGCGTGGAGCATTTCCCGCGAGTCCTGCAGTTTCTGCGCTCC
                                    ************************************************************

JH168072:139551-140968-TINF2-1      CTGCGCGCGGCCGCCCCCGGCTTGGTTCGCTACCGGCACCACGAGCGCTTGTGTATGGGC
JH168072:187433-188825-TINF2-2      CTGCGCGCCGCCGCCCCCGGCTTGGTTCGCTACCGGCGCCATGAGCGCTTGTGTATGGGC
                                    ******** ***************************** *** ******************

JH168072:139551-140968-TINF2-1      CTACAGGCCAAGGTATTGGAGCGCGGAGAATCTGGAAAGGGGCGGGGTGCGAGGACCTGT
JH168072:187433-188825-TINF2-2      CTACAGGCCAAGGTATTGGAGCGAG-------------------------GAGGACCTGT
                                    *********************** *                         **********

JH168072:139551-140968-TINF2-1      GGAGGGCTATCAGGTTGGGGGTGAGACTAGTTGGAAAGGGTCGCTTGCCCGAAACGGGCT
JH168072:187433-188825-TINF2-2      GGAGGGCTATCAGGTTGGGGGTGAGACTAGTTGGAAAGGGTCGCTTGCCCGAAACGGGCT
                                    ************************************************************

JH168072:139551-140968-TINF2-1      TCTGAGCTCAGTACCGTGTAGATGGTGGTGGAGATGATCCTGCAAGGCCAGCCTTGGGCT
JH168072:187433-188825-TINF2-2      TCTGAGCTCAGTACCGTGTAGATGGTGGTGGAGATGATCCTGCAAGGCCAGCCTTGGGCT
                                    ************************************************************

JH168072:139551-140968-TINF2-1      CAGGTTCTGAGTGTCCTGAATCGCCACTTCCCAGAGTCTGCACCTGCACCGCCCGACCCG
JH168072:187433-188825-TINF2-2      CAGGTTCTGAGTGTCCTGAATCGCCACTTCCCAGAGTCTGCACCTGCACCGCCCGACCCG
                                    ************************************************************

JH168072:139551-140968-TINF2-1      AGAGCTGTGAGTAATCCCTGGAACAAGCCCTAACCACAGTTACACTTAGTGCAACAAAGT
JH168072:187433-188825-TINF2-2      AGAGCTGTGAGTAATCCCTGGAACAAGCCCTAACCACAGTTACACTTAGTGCAACAAAGT
                                    ************************************************************

JH168072:139551-140968-TINF2-1      GGCCCCTTCTCCGGACCACTGGATCCTGCTGTCCCCAACTCTTATGCCTTCCATACCCTT
JH168072:187433-188825-TINF2-2      GGCCCCTTCTCCGGACCACTGGATCCTGCTGTCCCCAACTCTTATGCCTTCCATACCCTT
                                    ************************************************************

JH168072:139551-140968-TINF2-1      CCTCAACAGACAAAGCGGGATCTGAGGAAGATTTTGGAGGCACGGGAAACCTTTTGCCAG
JH168072:187433-188825-TINF2-2      CCTCAACAGACAAAGCGGGATCTGAGGAAGATTTTGGAGGCACGGGAAACCTTTTGCCAG
                                    ************************************************************

JH168072:139551-140968-TINF2-1      CACGTGAAGCAACTGTCAGAAGCCCCTGTGGATTTGGCCTCAAAGCTGCAGGTGAGACTG
JH168072:187433-188825-TINF2-2      CACGTGAAGCAACTGTCAGAAGCCCCTGTGGATTTGGCCTCAAAGCTGCAGGTGAGACTG
                                    ************************************************************

JH168072:139551-140968-TINF2-1      GCTGGTTTGCAGGTTATTAGGTGGCTCCTGTTTTTATCCTCCTCTTCTGACCTTCTTTTC
JH168072:187433-188825-TINF2-2      GCTGGTTTGCAGGTTATTAGGTGGCTCCTGTTTTTATCCTCCTCTTCTGACCTTCTTTTC
                                    ************************************************************

JH168072:139551-140968-TINF2-1      CACTCAGCAGGAGCTTGAACAAGAGTATGGGGATCCCTTTCTGGCAGCCATGGAAAAGCT
JH168072:187433-188825-TINF2-2      CACTCAGCAGGAGCTTGAACAAGAGTATGGGGATCCCTTTCTGGCAGCCATGGAAAAGCT
                                    ************************************************************

JH168072:139551-140968-TINF2-1      GTTTTTTGAATACTTGTGTCAGCTAGAGAAAGCACTGCCTGCAGTAGAGGCACAGCAGGT
JH168072:187433-188825-TINF2-2      GTTTTTTGAATACTTGTGTCAGCTAGAGAAAGCACTGCCTGCAGTAGAGGCACAGCAGGT
                                    ************************************************************

JH168072:139551-140968-TINF2-1      TTTGTTGGGGCAAAGCACAAGGGCAGAGTGTGGGGTGATTGTATCAAAAGCTGTGGTCTT
JH168072:187433-188825-TINF2-2      TTTGTTGGGGCAAAGCACAAGGGCAGAGTGTGGGGTGATTGTATCAAAAGCTGTGGTCTT
                                    ************************************************************

JH168072:139551-140968-TINF2-1      CATACATCTGCCTCCCCACAGCTTCAAGATGTGCTGAGTTGGATGCAGCCTGGAGTTTCT
JH168072:187433-188825-TINF2-2      CATACATCTGCCTCCCCACAGCTTCAAGATGTGCTGAGTTGGATGCAGCCTGGAGTTTCT
                                    ************************************************************

JH168072:139551-140968-TINF2-1      ATCACCTCTTCTCTTGCCTTGAGCCAGTATGCTGCAGACATGGGGTGGCCGCTTCCAGGT
JH168072:187433-188825-TINF2-2      ATCACCTCTTCTCTTGCCTTGAGCCAGTATGCTGCAGACATGGGGTGGCCGCTTCCAGGT
                                    ************************************************************

JH168072:139551-140968-TINF2-1      ACCAGGACCATCTGGCAACTAGCAATTTAGGGAGGTAGTGTTTAGCCTGACATCATCTGA
JH168072:187433-188825-TINF2-2      ACCAGGACCATCTGGCAACTAGCAATTTAGGGAGGTAGTGTTTAGCCTGACATCATCTGA
                                    ************************************************************
```

```
JH168072:139551-140968-TINF2-1      GTCAGCCCACTGGAAGTTACATGGGCCTGAAGAGTATCTGTTCTCTTTATAAGGAACCTC
JH168072:187433-188825-TINF2-2      GTCAGCCCACTGGAAGTTACATGGGCCTGAAGAGTATCTGTTCTCTTTATAAGGAACCTC
                                    ************************************************************

JH168072:139551-140968-TINF2-1      ATGTTTTTCTTTCAGATCCCTTTGTTTCTGGCTCAATGAACTTGACTGAGCCCATGGAAC
JH168072:187433-188825-TINF2-2      ATGTTTTTCTTTCAGATCCCTTTGTTTCTGGCTCAATGAACTTGACTGAGCCCATGGAAC
                                    ************************************************************

JH168072:139551-140968-TINF2-1      AGAATCATCCTCAGCAATCAAGACCAGCTCTCCACAGTCCTCTGCCAAAAGCCAAGCCCA
JH168072:187433-188825-TINF2-2      AGAATCATCCTCAGCAATCAAGACCAGCTCTCCACAGTCCTCTGCCAAAAGCCAAGCCCA
                                    ************************************************************

JH168072:139551-140968-TINF2-1      GCCCTCAGGGACCAGCTTCAAGGAAGCATCCCGAATCTTTAGCTGGCCACCACTTCAATC
JH168072:187433-188825-TINF2-2      GCCCTCAGGGACCAGCTTCAAGGAAGCATCCCGAATCTTTAGCTGGCCACCACTTCAATC
                                    ************************************************************

JH168072:139551-140968-TINF2-1      TGGCCCCGCTAGGCCAGCGAAAACTCCAGTCCCGATGGACATCTGCAACGGGCAGCTATA
JH168072:187433-188825-TINF2-2      TGGCCCCGCTAGGCCAGCGAAAACTCCAGTCCCGATGGACATCTGCAACGGGCAGCTATA
                                    ************************************************************

JH168072:139551-140968-TINF2-1      AAGAGCGCCCCACGGTCATGCTGTTGCCCTTCAGGAATNNNNNNNNNNNNNNNNNNNNNN
JH168072:187433-188825-TINF2-2      AAGAGCGCCCCACGGTCATGCTGTTGCCCTTCAGGAATNNNNNNNNNNNNNNNNNNNNNN
                                    ************************************************************

JH168072:139551-140968-TINF2-1      NNNNNNNNNNNNNNNNNNNNNNNNNNNNNNNNNNNNNNNNNNNNNNNNNNNNNNNNNNNN
JH168072:187433-188825-TINF2-2      NNNNNNNNNNNNNNNNNNNNNNNNNNNNNNNNNNNNNNNNNNNNNNNNNNNNNNNNNNNN
                                    ************************************************************

JH168072:139551-140968-TINF2-1      NNNNNNNNNNNNNNNNNNNNNNNNNNNNNNNNNNNNNNNNNNNNNNNNNNNNNNNNNNNN
JH168072:187433-188825-TINF2-2      NNNNNNNNNNNNNNNNNNNNNNNNNNNNNNNNNNNNNNNNNNNNNNNNNNNNNNNNNNNN
                                    ************************************************************

JH168072:139551-140968-TINF2-1      NNNNNNNNNNNNNNNNNNNNNNNNNNNNNNNNNNNNNNNNNNNNNNNNNNNNNNNNNNNN
JH168072:187433-188825-TINF2-2      NNNNNNNNNNNNNNNNNNNNNNNNNNNNNNNNNNNNNNNNNNNNNNNNNNNNNNNNNNNN
                                    ************************************************************

JH168072:139551-140968-TINF2-1      NNNNNNNNNNNNNNNNNNNNNNNNNNNNNNNNNNNNNNNNNNNNNNNNNNNNNNNNNNNN
JH168072:187433-188825-TINF2-2      NNNNNNNNNNNNNNNNNNNNNNNNNNNNNNNNNNNNNNNNNNNNNNNNNNNNNNNNNNNN
                                    ************************************************************

JH168072:139551-140968-TINF2-1      NNNNNNNNNNNNNNNNNNNNNNNNNNNNNNNNNNNNNNNNNNNNNNNNNNNNNNNNNNNN
JH168072:187433-188825-TINF2-2      NNNNNNNNNNNNNNNNNNNNNNNNNNNNNNNNNNNNNNNNNNNNNNNNNNNNNNNNNNNN
                                    ************************************************************

JH168072:139551-140968-TINF2-1      NNNNNNNNNNNNNNNNNNNNNNNNNNNNNNNNNNNNNNNNNNNNNNNNNNNNNNNNNNNN
JH168072:187433-188825-TINF2-2      NNNNNNNNNNNNNNNNNNNNNNNNNNNNNNNNNNNNNNNNNNNNNNNNNNNNNNNNNNNN
                                    ************************************************************

JH168072:139551-140968-TINF2-1      NNNNNNNNNNNNNNNNNNNNNNNNNNNNNNNNNNNNNNNNNNNNNNNNNNNNNNNNNNNN
JH168072:187433-188825-TINF2-2      NNNNNNNNNNNNNNNNNNNNNNNNNNNNNNNNNNNNNNNNNNNNNNNNNNNNNNNNNNNN
                                    ************************************************************

JH168072:139551-140968-TINF2-1      NNNNNNNNNNNNNNNNNNNNNNNNNNNNNNNNNNNNNNNNNNNNNNNNNNNNNNNNNNNN
JH168072:187433-188825-TINF2-2      NNNNNNNNNNNNNNNNNNNNNNNNNNNNNNNNNNNNNNNNNNNNNNNNNNNNNNNNNNNN
                                    ************************************************************

JH168072:139551-140968-TINF2-1      NNNNNNNNNNNNNNNNNNNNNNNNNNNNNNNNNNNNNNNNNNNNNNNNNNNNNNNNNNNN
JH168072:187433-188825-TINF2-2      NNNNNNNNNNNNNNNNNNNNNNNNNNNNNNNNNNNNNNNNNNNNNNNNNNNNNNNNNNNN
                                    ************************************************************

JH168072:139551-140968-TINF2-1      NNNNNNNNNNNNNNNNNNNNNNNNNNNNNNNNNNNNNNNNNNNNNNNNNNNNNNNNNNNN
JH168072:187433-188825-TINF2-2      NNNNNNNNNNNNNNNNNNNNNNNNNNNNNNNNNNNNNNNNNNNNNNNNNNNNNNNNNNNN
                                    ************************************************************

JH168072:139551-140968-TINF2-1      NNNNNNNNNNNNNNNNNNNNNNNNNNNNNNNNNNNNNNNNNNNNNNNNNNNNNNNNNNNN
JH168072:187433-188825-TINF2-2      NNNNNNNNNNNNNNNNNNNNNNNNNNNNNNNNNNNNNNNNNNNNNNNNNNNNNNNNNNNN
                                    ************************************************************

JH168072:139551-140968-TINF2-1      NNNNNNNNNNNNNNNNNNNNNNNNNNNNNNNNNNNNNNNNNNNNNNNNNNNNNNNNNNNN
JH168072:187433-188825-TINF2-2      NNNNNNNNNNNNNNNNNNNNNNNNNNNNNNNNNNNNNNNNNNNNNNNNNNNNNNNNNNNN
                                    ************************************************************

JH168072:139551-140968-TINF2-1      NNNNNNNNNNNNNNNNNNNNNNNNNNNNNNNNNNNNNNNNNNNNNNNNNNNNNNNNNNNN
JH168072:187433-188825-TINF2-2      NNNNNNNNNNNNNNNNNNNNNNNNNNNNNNNNNNNNNNNNNNNNNNNNNNNNNNNNNNNN
                                    ************************************************************

JH168072:139551-140968-TINF2-1      NNNNNNNNNNNNNNNNNNNNNNNNNNNNNNNNNNNNNNNNNNNNNNNNNNNNNNNNNNNN
JH168072:187433-188825-TINF2-2      NNNNNNNNNNNNNNNNNNNNNNNNNNNNNNNNNNNNNNNNNNNNNNNNNNNNNNNNNNNN
                                    ************************************************************
```

```
JH168072:139551-140968-TINF2-1      NNNNNNNNNNNNNNNNNNNNNNNNNNNNNNNNNNNNNNNNNNNNNNNNNNNNNNNNNNNN
JH168072:187433-188825-TINF2-2      NNNNNNNNNNNNNNNNNNNNNNNNNNNNNNNNNNNNNNNNNNNNNNNNNNNNNNNNNNNN
                                    ************************************************************

JH168072:139551-140968-TINF2-1      NNNNNNNNNNNNNNNNNNNNNNNNNNNNNNNNNNNNNNNNNNNNNNNNNNNNNNNNNNNN
JH168072:187433-188825-TINF2-2      NNNNNNNNNNNNNNNNNNNNNNNNNNNNNNNNNNNNNNNNNNNNNNNNNNNNNNNNNNNN
                                    ************************************************************

JH168072:139551-140968-TINF2-1      NNNNNNNNNNNNNNNNNNNNNNNNNNNNNNNNNNNNNNNNNNNNNNNNNNNNNNNNNNNN
JH168072:187433-188825-TINF2-2      NNNNNNNNNNNNNNNNNNNNNNNNNNNNNNNNNNNNNNNNNNNNNNNNNNNNNNNNNNNN
                                    ************************************************************

JH168072:139551-140968-TINF2-1      NNNNNNNNNNNNNNNNNNNNNNNNNNNNNNNNNNNNNNNNNNNNNNNNNNNNNNNNNNNN
JH168072:187433-188825-TINF2-2      NNNNNNNNNNNNNNNNNNNNNNNNNNNNNNNNNNNNNNNNNNNNNNNNNNNNNNNNNNNN
                                    ************************************************************

JH168072:139551-140968-TINF2-1      NNNNNNNNNNNNNNNNNNNNNNNNNNNNNNNNNNNNNNNNNNNNNNNNNNNNNNNNNNNN
JH168072:187433-188825-TINF2-2      NNNNNNNNNNNNNNNNNNNNNNNNNNNNNNNNNNNNNNNNNNNNNNNNNNNNNNNNNNNN
                                    ************************************************************

JH168072:139551-140968-TINF2-1      -------------------------
JH168072:187433-188825-TINF2-2      NNNNNNNNNNNNNNNNNNNNNNNNN
```

Supplementary Figure 6. Alignment of RNA-seq reads to two copies of TINF2 gene in NMR shows expression in both copies.

REFERENCES

Birney, E., Clamp, M., and Durbin, R. (2004). GeneWise and Genomewise. Genome Res *14*, 988-995.

Edgar, R.C. (2004). MUSCLE: multiple sequence alignment with high accuracy and high throughput. Nucleic acids research *32*, 1792-1797.

Fang, X., Seim, I., Huang, Z., Gerashchenko, M.V., Xiong, Z., Turanov, A.A., Zhu, Y., Lobanov, A.V., Fan, D., Yim, S.H.*, et al.* (2014). Adaptations to a Subterranean Environment and Longevity Revealed by the Analysis of Mole Rat Genomes. Cell reports.

Keane, M., Craig, T., Alfoldi, J., Berlin, A.M., Johnson, J., Seluanov, A., Gorbunova, V., Di Palma, F., Lindblad-Toh, K., Church, G.M.*, et al.* (2014). The Naked Mole Rat Genome Resource: facilitating analyses of cancer and longevity-related adaptations. Bioinformatics.

Kearse, M., Moir, R., Wilson, A., Stones-Havas, S., Cheung, M., Sturrock, S., Buxton, S., Cooper, A., Markowitz, S., Duran, C.*, et al.* (2012). Geneious Basic: an integrated and extendable desktop software platform for the organization and analysis of sequence data. Bioinformatics *28*, 1647-1649.

Kent, W.J. (2002). BLAT--the BLAST-like alignment tool. Genome research *12*, 656-664.

Kent, W.J., Sugnet, C.W., Furey, T.S., Roskin, K.M., Pringle, T.H., Zahler, A.M., and Haussler, D. (2002). The human genome browser at UCSC. Genome Res *12*, 996-1006.

Kim, E.B., Fang, X., Fushan, A.A., Huang, Z., Lobanov, A.V., Han, L., Marino, S.M., Sun, X., Turanov, A.A., Yang, P.*, et al.* (2011). Genome sequencing reveals insights into physiology and longevity of the naked mole rat. Nature *479*, 223-227.

Kimura, M. (1980). A simple method for estimating evolutionary rates of base substitutions through comparative studies of nucleotide sequences. Journal of molecular evolution *16*, 111-120.

Li, H., and Durbin, R. (2009). Fast and accurate short read alignment with Burrows-Wheeler transform. Bioinformatics *25*, 1754-1760.

Li, W.H. (1993). Unbiased estimation of the rates of synonymous and nonsynonymous substitution. Journal of molecular evolution *36*, 96-99.

Ostlund, G., Schmitt, T., Forslund, K., Kostler, T., Messina, D.N., Roopra, S., Frings, O., and Sonnhammer, E.L. (2010). InParanoid 7: new algorithms and tools for eukaryotic orthology analysis. Nucleic acids research *38*, D196-203.

Wu, T.D., and Watanabe, C.K. (2005). GMAP: a genomic mapping and alignment program for mRNA and EST sequences. Bioinformatics *21*, 1859-1875.
